# Probing the Behaviour of Fluids Confined in Porous Materials by Neutron Scattering: Applications to CO_2_ Sequestration and Enhanced Oil and Gas Recovery

**DOI:** 10.1002/cplu.202400353

**Published:** 2024-10-24

**Authors:** Konstantinos L. Stefanopoulos

**Affiliations:** ^1^ Institute of Nanoscience and Nanotechnology National Centre for Scientific Research “Demokritos” 153 10 Ag. Paraskevi Athens Greece

**Keywords:** Confined fluids, Neutron scattering, CO_2_ sequestration, Enhanced oil recovery, Porous materials

## Abstract

The current review presents a discussion on the utility of neutron scattering, with emphasis on neutron total scattering and small‐angle neutron scattering (SANS), to explore the structural properties and the phase behaviour of fluids confined in nanopores. The effectiveness of *contrast matching* SANS on the evaluation of accessibility of porous materials to invading fluids is highlighted too. This review provides also an overview regarding the neutron scattering studies on the structure and the accessibility of greenhouse gases in the complex pore network of geomaterials, with applications to CO_2_ geological sequestration and enhanced oil and gas recovery.

## Graphical abstract

Understanding the properties of confined fluids is a challenging research area and a key feature for many applications. The review summarises the utility of neutron scattering for the structural investigation of fluids confined in porous materials. The review highlights advances in this area, particularly focusing on greenhouse gases confined in geological materials with applications to CO_2_ sequestration and enhanced oil and gas recovery.

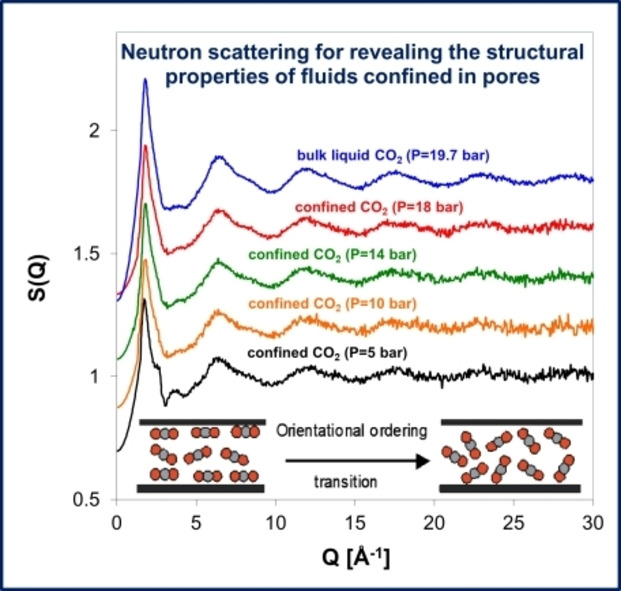

## 1 Introduction

The structural and thermodynamic properties of bulk fluids are nowadays well investigated and understood. When fluids are in confinement, however, the combination of surface‐fluid interactions, as well as the finite volume, can significantly alter their structural and thermodynamic properties and strongly affect their phase behaviour. Especially, when confined in nanoscale pores, several factors such as pore size distribution, interconnectivity of pores and the surface chemistry can influence their properties. In this perspective, the study of confined fluids remains an open and challenging research area because the existing measurements and equations of state for bulk fluids are not applicable. Apart from the theoretical point of view, a further understanding of the properties of confined fluids is a key feature for various applications, such as capture and sequestration of anthropogenic greenhouse gases, oil and gas recovery, hydrogen storage, catalysis, gas separation, chromatography, super capacitors and many others.[^1^, ^2^, ^3^, ^4^, ^5^]

Neutron scattering is a powerful tool to probe *non‐destructively* the *structure* and the *dynamics* of fluids confined in porous materials, as well as the structural properties of pores. In general, both neutron scattering theory[^6^, ^7^] and applications[^8^, ^9^, ^10^] are well‐documented. X‐ray scattering is also widely used to evaluate the properties, interactions and structures of porous materials and fluids. However, there are differences between these methods. X‐rays interact with matter through electromagnetic interactions with the electron cloud of atoms, while neutrons interact weakly with nuclei via nuclear forces. The fact that nucleus is only 0.1 % of the diameter of the atom means that most of matter is empty space and consequently neutrons are highly penetrating. Furthermore, neutrons are uncharged and this is the other reason that they can penetrate deeply into the sample. The advantage of the high penetration capability of neutrons over X‐rays is that neutron experiments provide information mainly about the bulk of the sample rather than its surface. For X‐rays, typical sample thickness varies from 0.01 to 1 mm, whereas neutron samples range can vary between 0.5 and 5 mm. Finally, the neutron penetration capability makes then easier to utilise advanced sample environment such as gas handling equipment, cryostats, furnaces, magnets and high‐pressure sample containers for monitoring *in situ* various processes in porous materials, such as adsorption, flow, catalytic reactions, oil and gas recovery, etc.

Furthermore the atomic scattering factor for X‐rays is proportional to the number of electrons, increases monotonically with atomic number (Z) and is identical for the isotopes of the same element. This is not the case for the neutron coherent scattering length, *b*, that varies rather randomly with atomic number and allows not only the distinction between two neighbouring elements in the periodic table but also between different isotopes of the same element (such as hydrogen and deuterium, which have scattering lengths of opposite sign).^[11]^

On the other hand, the main disadvantages of neutron scattering are the relatively low neutron flux compared to X‐rays and, thus, the requirement of much longer data acquisition time. Moreover, neutrons are difficult to produce and they are expensive. Consequently, while X‐ray‐based facilities (such as synchrotron) can be more easily accessed, getting access to the few neutron facilities worldwide (reactors or accelerators) is neither an easy nor a quick procedure. The reason is that the beamtime for neutrons is allocated on a very competitive basis after a peer‐review process, in which experimental proposals are evaluated by scientific panels commonly twice a year.

Neutron scattering can be categorised into *elastic* and *inelastic*. Elastic neutron scattering (also known as neutron diffraction) involves processes, in which there is no energy loss (but only change to direction upon collision) between the incident and the scattered neutrons. Elastic neutron scattering techniques include neutron diffraction, neutron total scattering, small‐angle neutron scattering (SANS), ultra‐small‐angle neutron scattering (USANS), neutron reflectometry and spin‐echo small‐angle neutron scattering (SESANS). In addition, neutron logging and imaging are other techniques that use neutron scattering.

*Neutron powder diffraction* is a powerful tool for the structural study of crystalline porous materials, especially when they contain light atoms (such as H, D, Li, C, N, O). The reason is that light elements may be poorly elucidated in a sample investigated by X‐ray diffraction, especially when the sample consists also of elements with high atomic number, Z. Another advantage of neutron diffraction is that the Bragg reflections have similar intensities over the whole pattern, while in X‐ray diffraction patterns the intensity of Bragg peaks decreases with the increasing 2*θ* scattering angle.^[12]^ For instance, by utilising neutron diffraction, a lot of knowledge can be gained about both the location and the interaction of guest molecules within complex crystalline porous frameworks (such as MOFs). It is worth mentioning that the diffraction method is mainly used to determine the phase and precise atomistic information of crystalline materials, while the total scattering method examines the local atomic structure of systems that exhibit short‐rang order and they do not have well‐defined Bragg diffraction peaks, such as amorphous and disordered materials. As a result, neutron total scattering is a very promising technique for the structural study of confined fluids. Noteworthy, neutron total scattering is not always differentiated from neutron diffraction in the literature.

Complementary to neutron diffraction (and total scattering) that measures interatomic distances, SANS and USANS (or (U)SANS) probe the structures at larger length scales (from 1 nm up to few micrometers) and they are essential tools to reveal the structure of porous materials, including also materials with complex pore architecture such as sedimentary rocks. Low‐pressure gas (N_2_, CO_2_) physisorption by volumetric or gravimetric methods and mercury intrusion porosimetry are also conventional methods which routinely used for investigating the structural properties of the pore network in porous solids such as pore volume, pore size distribution, surface area etc. Their main limitation, however, is that they are invasive providing, thus, information only about the accessible (open) pores. When (U)SANS, however, is combined with adsorption or *contrast matching* SANS, valuable information can be gained not only about the structural properties and the phase behaviour of confined fluids but also about the *accessibility* of pores to various fluids. The reason is that neutrons have the ability to “*see but not destruct*” the pores and they can also “*monitor*” the changes in the sorption behaviour of fluid molecules in a much broader length scale and topology. Further, neutrons have also the unique ability to “*detect*” the pores that are inaccessible to the invading fluid (closed pores), which cannot be measured by other techniques. Conclusively, each of these methods is valuable and complementary, particularly for materials with complex pore network and closed porosity like sedimentary rocks.[^13^, ^14^, ^15^, ^16^]

In inelastic neutron scattering (INS) or neutron spectroscopy, neutrons can gain or lose energy in the scattering process. This energy transfer appears as the scatterer's rotational, vibrational, or translational energy. Neutron spectroscopy, which includes time‐of‐flight scattering, neutron triple‐axis spectrometry, neutron backscattering and neutron spin echo techniques, is widely used to study atomic vibrations and other excitations. INS is highly complementary with Raman/infrared spectroscopy, but it has great sensitivity to hydrogen (H). It is also worth mentioning that there is a special type of scattering called *quasi‐elastic neutron scattering* (QENS), a powerful tool for diffusion studies, which provides information about the mobility of the pore‐confined fluid (translation and rotation motions) on a microscopic scale from few Ångstrom to few nanometers.[^17^, ^18^, ^19^] Noteworthy, QENS is complementary to nuclear magnetic resonance (NMR) spectroscopy.[^20^, ^21^]

*Carbon capture and storage* (CCS) has been recognised key technology to reduce greenhouse gas emissions from fossil fuel‐based power plants and industrial sources in an attempt to limit the effects of global warming and climate change. CCS separates CO_2_ from other gases released by industrial processes and transports the captured CO_2_ to storage. There are several methods of carbon storage, including geological storage, ocean storage, and mineral storage. Geological storage is the most popular method and involves in the CO_2_ injection into subterranean geological formations like coal seams, deep saline formations and depleted oil and gas reserves.^[22]^ Although CCS plays a key role in preventing CO_2_ from entering the atmosphere and contributing to global warming, there are still technical and operational challenges associated with the high operation cost of CCS technology and the need for public acceptance of CCS as a viable solution for climate change.

CCS can also be used as an alternative for water injection into oil reservoirs, a technique known as carbon capture, utilisation, and storage (CCUS) or enhanced oil recovery (EOR). This involves injecting CO_2_ into oil and gas reservoirs, which helps to reduce the viscosity of the oil and making it easier to extract, while CO_2_ is permanently stored underground.^[23]^ Alternatively, the use of supercritical CO_2_ for exploitation of natural gas from unconventional shale reservoirs has emerged as a better option compared to water‐based fracturing fluids. The CO_2_ injection can then be applied to enhance shale gas recovery (EGR) with the objectives of drilling, fracturing and sequestration.[^24^, ^25^] Furthermore, the geological sequestration of CO_2_ in coal seams holds significant implications for enhanced coalbed methane (ECBM) development and greenhouse gas mitigation.^[26]^ Overall, the oil and gas industry could benefit from the utility of carbon storage methods, such as CO_2_ geological sequestration, aiming simultaneously to decrease the carbon footprint and enhance the recovery. Another benefit of oil and gas recovery projects is that, after the project completion, the same place could be used without any additional investment for further CO_2_ sequestration.^[27]^ Nowadays, CO_2_‐EOR provides the largest market demand for carbon dioxide with the potential to generate revenue from the production of over 1000 billion barrels of oil, while storing over 300 Gt CO_2_ worldwide.^[28]^ It is worth mentioning that experts claim that the pore space in sedimentary rocks available globally is more than enough to sequester all the CO_2_ that humanity could ever want to remove from the air.^[29]^

Sedimentary rocks (such as coal, shale, sandstone, carbonate) are formed by the deposition or accumulation of organic particles or mineral at earth's surface, followed by cementation. They have a complex pore network characterised by an extremely wide pore size distribution including all types of pores, extending from the subnanometer (micropores) to micron (macropores) length scale.^[30]^ Carbonates or sandstones are commonly found in hydrocarbon oil and gas reservoirs, while organic‐rich shales are hydrocarbon source rocks and in some cases they are also reservoir rocks. Finally, coals can also be source rocks for oil and gas (coalbed methane, or CBM), although they are energy resource on their own. These rock formations are composed of complex and chemically heterogeneous pore networks and contain other chemical species like water and hydrocarbons. Advanced non‐invasive and multi‐scale methods such as SANS and USANS techniques, combined with traditional porosimetry methods have been extensively utilised for probing the rock microstructure and porosity, as they play a crucial role in many geological processes including CO_2_ sequestration and oil and gas recovery. (U)SANS methods have also revealed that sedimentary rocks display the most extensive fractal behaviour observed in nature, with self‐similarity extending over at least three orders of magnitude in the length scale.^[31]^ Moreover, *contrast matching* (U)SANS is a unique tool for estimating the *pore accessibility* of various sedimentary rocks to greenhouse gases, such as CO_2_ and CH_4_, which is a key parameter for CO_2_ sequestration and oil/gas recovery. The methane accessibility to pores of unconventional coal and shale reservoirs is another critical factor, which affects both the methane storage capacity and methane desorption. As a result, the evaluation of closed or isolated pores is of great importance because they may contain methane which could influence both the CO_2_ sequestration and the enhanced gas recovery. Furthermore, neutron total scattering combined with (U)SANS can reveal both the structural properties and the phase behaviour of greenhouse fluids in confined environments. They can also be utilised to monitor processes like enhanced oil recovery in real time at the nanoscale. Conclusively, an in‐depth understanding of pore accessibility and structure of greenhouse fluids confined within the complex pore network of sedimentary rocks is of great importance for the achievement of optimal CO_2_ sequestration, as well as enhanced oil and gas recovery.

The current review study will focus on the elucidation of structural properties and phase behaviour of fluids confined in porous materials by neutron total scattering and (U)SANS. In particular, Section 2 describes the basic principles of neutron total scattering on confined fluids and reviews the main advances on this area. Section 3 briefly introduces the (U)SANS technique, focusing on the effectiveness of *contrast matching* (U)SANS method on the investigation of accessibility of porous materials to invading fluids. Furthermore, *in situ* gas adsorption and SANS studies in porous materials, as well as SANS applications to pore‐confined ionic liquids are revised. Section 4 deals with the application of neutron total scattering and (U)SANS to CO_2_ sequestration and enhanced oil and gas recovery. In particular, it discusses the confinement effects of greenhouse gases (especially CO_2_ and CH_4_) within the nanopores of sedimentary rocks (mainly coal and shale), as well the pore accessibility of these geomaterials to greenhouse gases.

## 2 Neutron Total Scattering from Confined Fluids

Neutron diffraction is nowadays routinely used to determine both the phase and the atomistic structure of perfectly ordered *crystalline materials*. Both X‐ray and neutron diffraction experiments usually consider only the *Bragg scattering* to reveal the long‐range order of the atomic structure, represented by the unit cell. However, in practice, the atomic arrangement in real crystals exhibits always deviations from the ideal periodicity due to thermal vibrations, dislocations etc. This type of “*weak disorder*” gives rise to *diffuse scattering* resulting in a reduction in the Bragg scattering. When only Bragg scattering is analysed, commonly by using Rietveld refinement, the diffuse scattering is simply assumed as background. *Disordered materials* however, such as liquids, glasses, polymers and various gels, films and porous solids, exhibit short‐range or limited‐range crystalline structure. As a result, all the correlations between the disordered structural features are contained in the diffuse scattering. The structural information can therefore be provided only by the measurement of the *total scattering* (i. e. Bragg scattering and diffuse scattering).

**Figure 1:**
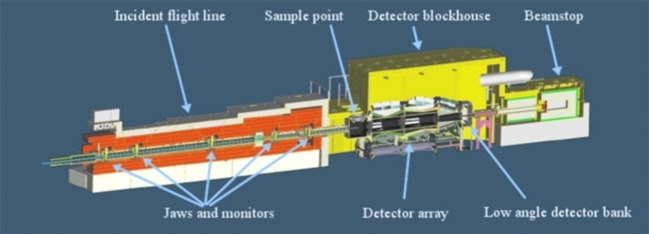
Cutaway diagram of NIMROD giving an overview of the key components from which it is constructed. NIMROD is located at TS2, ISIS Neutron and Muon Source, UKRI Science and Technology Facilities Council (Oxfordshire, UK). Reproduced from Ref. [38] with permission from AIP Publishing.

In principle, there is no difference between a neutron total scattering instrument and a neutron powder diffractometer (neutron total scattering is often referred as neutron diffraction in the literature). They are both used to measure the scattered intensity as a function of the *scattering vector* or the *momentum transfer*, *Q*.^[32]^ However, for total scattering measurements, the instrument should have the capability to collect data over a very large *Q* range with sufficient resolution, in order to achieve an effective radial distribution function analysis (Figure 1). In addition, significant neutron flux and low background are also required in order to measure the weak signal from diffuse scattering with appropriate statistics. Finally, neutron total scattering has been proved to be a powerful tool, compared to X‐rays, for the structural investigation of disordered systems containing light elements. For an in‐depth information about total scattering methodology, I would suggest the book by Egami & Billinge,^[33]^ as well as the reviews by Dove & Li,^[34]^ by Keen^[35]^ and by Gelisio & Scardi.^[36]^ Keen^[37]^ also explains explicitly the meaning of different definitions of various commonly used correlation functions. In the following, I will discuss about the basic principles of neutron total scattering, focused on the study of confined fluids.

In general, the raw neutron data obtained from a total scattering experiment are subject to standard corrections for background scattering, multiple scattering, container scattering and self‐attenuation; furthermore, the data are normalised on absolute scale of *differential scattering cross section (DCS) per atom of material* by using a standard vanadium scatterer. DCS is defined as follows:^[35]^

(1) $$\frac{d\sigma }{d\Omega }=\sum_{i}c_{i}b_{i}^{2}+F\left(Q\right)$$

where *Q* is the scattering vector and *c_i_*, *b_i_*, are the atomic fraction and neutron coherent scattering length of atom *i*, respectively. *F*(*Q*) is the *total structure factor* and $4\pi \sum_{i}c_{i}b_{i}^{2}$ is the neutron *total scattering cross section* of the material. The *total structure factor* or the *interference scattering* of a disordered system is originating from intra‐ and intermolecular correlations and it involves a sum over pairs of atom types:^[38]^

(2) $$F\left(Q\right)=\sum_{\alpha \leq \beta }\left(2-\delta_{\alpha \beta }\right)c_{\alpha }c_{\beta }b_{\alpha }b_{\beta }\left[S_{\alpha \beta }\left(Q\right)-1\right]$$

where *Q* is the scattering vector, *c*_*α*_ and *c*_*β*_ are the concentrations of atom types *α* and *β* in the sample and *b*_α_ and *b*_*β*_ are their corresponding neutron coherent scattering lengths. *S*_*αβ*_(Q) are the partial structure factors corresponding to the pairwise correlations between atoms of type *α* and *β*; *δ*_*αβ*_ is the Kronecker delta function to avoid double counting interactions between like‐atom pairs.^[39]^ The total structure factor can be inverted by Fourier transform to the *total radial distribution function*, *g*(*r*), weighted by the atomic density, *ρ*, of the system:^[38]^

(3) $$g\left(r\right)-1=\frac{1}{{\left(2\pi \right)}^{3}\rho }\underset{0}{\int^{\infty }}4\pi Q^{2}F\left(Q\right)\frac{sinQr}{Qr}dQ$$

In principle, *g*(*r*), is the weighted probability that atoms might be found at certain distances from other atoms. It can be seen that the upper *Q* limit on the integral is critical for the resolution of the radial distribution function; this is the reason why a total scattering instrument should have the ability to measure large values of *Q*.^[40]^ It should be also noted that another useful function is the *total differential pair correlation function*, *D*(*r*), which emphasises the long‐range correlations more prominently by weighting them by the length scale on which they occur, without masking the short‐range structure:^[38]^

(4) $$D\left(r\right)=4\pi \rho r\left[g\left(r\right)-1\right]$$

For a bulk molecular liquid, the *molecular structure factor*, *S_M_* (*Q*), can be written:^[41]^

(5) $$S_{M}\left(Q\right)=f_{1}\left(Q\right)+D_{M}\left(Q\right)=\;f_{1}\left(Q\right)+\frac{4\pi }{Q}\rho_{M}\int \left[g\left(r\right)-1\right]rsin\left(Qr\right)dr$$

where *f_1_* (*Q*) is the intramolecular form factor and *D_M_* (*Q*) is the intermolecular contribution which contains all the structural information of the liquid; *ρ*_*Μ*_ is the physical density of the liquid.

When a fluid is confined in a porous matrix, the structure factor arises from the superposition of the matrix‐matrix and fluid‐fluid correlations themselves, as well as from the *cross correlation* between the matrix and the fluid. The contribution of the matrix‐matrix correlations are contained in the empty matrix and can be properly subtracted from the experimental data. As a result, the total structure factor of the confined fluid reduces in the following two terms:[^42^, ^43^]

(6) $$S\left(Q\right)=S_{M}^{\mathrm{Fluid}}\left(Q\right)+2\sqrt{\frac{X_{\mathrm{Matrix}}}{X_{\mathrm{Fluid}}}}\frac{b_{\mathrm{Matrix}}}{b_{\mathrm{Fluid}}}S_{M}^{\mathrm{Matrix}-\mathrm{Fluid}}\left(Q\right)$$

where$\;S_{M}^{\mathrm{Fluid}}\left(Q\right)$ is the structure factor of the bulk fluid (see Equation (5)), $S_{M}^{\mathrm{Matrix}-\mathrm{Fluid}}\left(Q\right)$ is the *cross correlation* between the solid matrix and the fluid; $b_{\mathrm{Matrix}}$ ,$\;b_{\mathrm{Fluid}}$ , $X_{\mathrm{Matrix}}$ , $X_{\mathrm{Fluid}}$ are the coherent scattering lengths and the mole fractions of the solid matrix and the fluid, respectively.^[44]^ It is worth mentioning that the total structure factor is also affected by the *exclusion volume* effect induced by the requirement that a fraction of space, outside of the pore volume, is inaccessible to the fluid molecules.^[45]^

**Figure 2:**
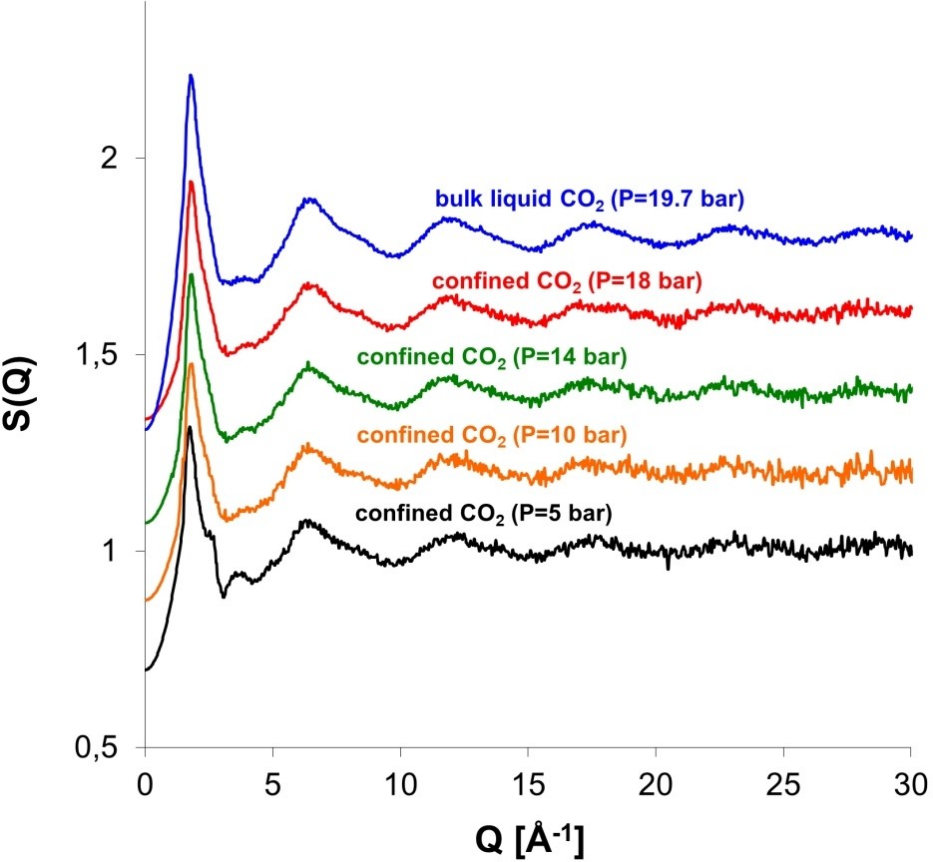
The total scattering structure factor at 253 K for adsorbed CO_2_ at 5 bar (monolayer coverage), at 10, 14 and 18 bar (corrected for cross correlation term on the basis of monolayer coverage) and for the bulk liquid at 19.7 bar (see text, for details). The structure factors have shifted by 0.2 for clarity. Note the elimination of the shoulder *Q*=2.7 Å^−1^ for the confined CO_2_ at 10, 14 and 18 bar, respectively. Reproduced and modified from Ref. [46] with permission from APS.

**Figure 3:**
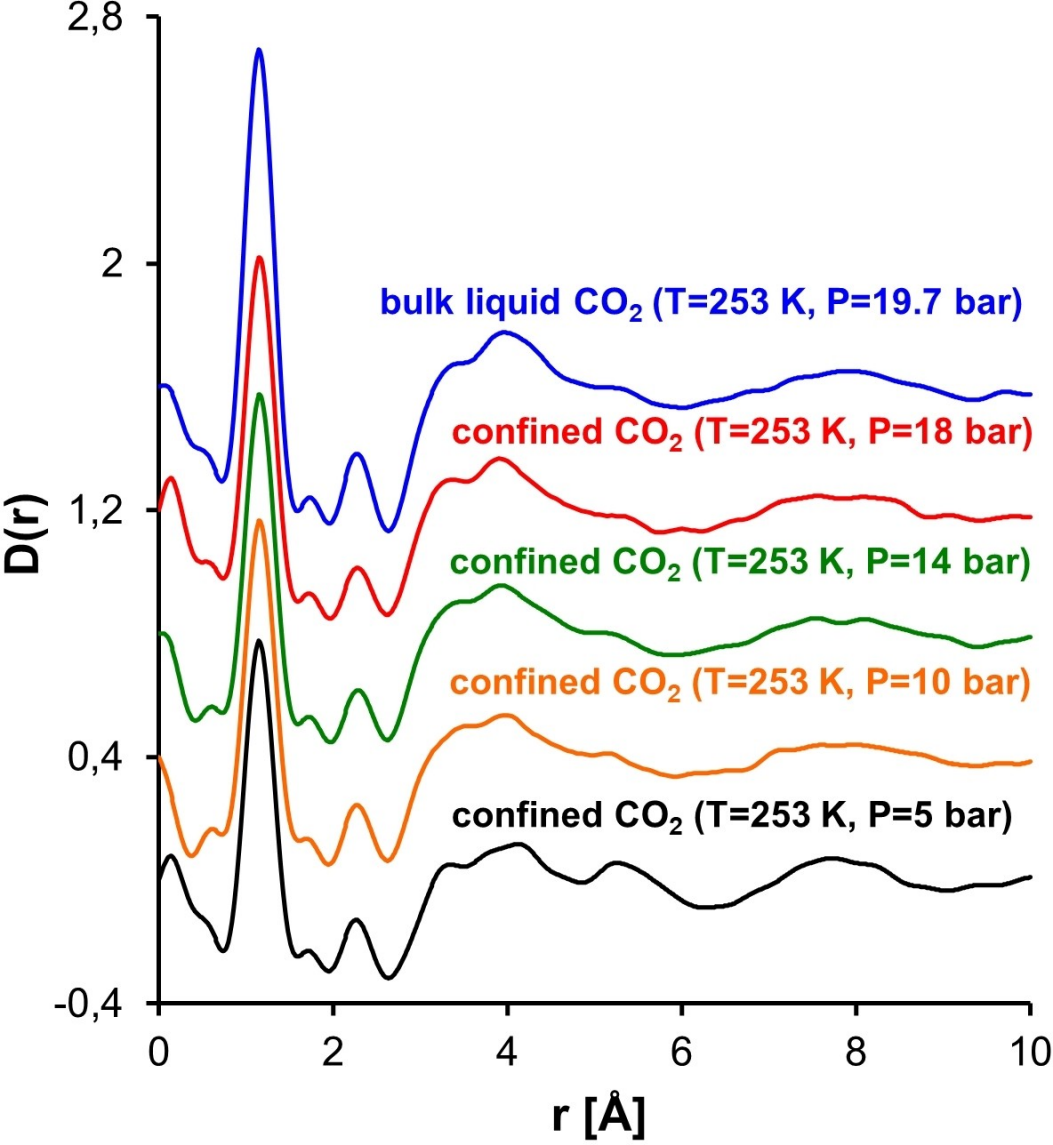
The differential correlation function at 253 K for adsorbed CO_2_ at 5 bar (monolayer coverage), at 10, 14 and 18 bar (corrected for cross correlation term on the basis of monolayer coverage) and for the bulk liquid at 19.7 bar (see text, for details). The correlation functions have shifted by 0.2 for clarity. Reproduced and modified from Ref. [46] with permission from APS.

When sorption and neutron total scattering are combined the structural properties of the confined phase can be elucidated during pore filling. Steriotis et al.^[46]^ carried out *in situ* CO_2_ adsorption along an isotherm at 253 K in ordered mesoporous MCM‐41 and neutron total scattering measurements. Noteworthy, ordered mesoporous silica materials have drawn much attention for various applications such as gas storage, heterogeneous catalysis, separation processes and drug delivery. MCM‐41 consists of a hexagonal array of cylindrical pores with size about 3 nm. The experimental total structure factors and the total differential pair correlation functions of sorbed subcritical CO_2_ have been evaluated during various stages of pore filling and compared to the bulk liquid (Figure 2 and Figure 3).

In general, the total structure factor of a liquid consists of the *intermolecular part* (between the molecules) at the low‐*Q* regime and the *intramolecular part* (within the molecule) at the large *Q* region. The *intermolecular structure peak* corresponds to the most probable distance between nearest‐neighbour molecules. The intermolecular structure peak of bulk liquid CO_2_ is located at about 1.79 Å^−1^ followed by five long‐range oscillations at larger *Q*s, which correspond to intramolecular correlations (Figure 2). The main peak possesses a degree of asymmetry, exhibits a minimum at Q~3 Å^−1^ and a tiny bump near 4 Å^−1^ (Figure 2). The main peak asymmetry can be explained in terms of the orientational correlations between neighbouring carbon dioxide molecules which arise mainly from electrical quadrupolar interactions. The structure factors from confined CO_2_ present also a main peak followed by intramolecular oscillations, although less pronounced. It is also clearly observed that, during pore filling, the peak positions shift slightly to higher *Q* values and reach that of the bulk liquid at pressures 14 and 18 bar, respectively. At these pressures, capillary condensation has occurred and the pores are completely filled with CO_2_ (Figure 2). The calculated density of the confined CO_2_ obtained from the adsorption isotherm for the saturated pores was similar to that of bulk liquid (~1 g/cm^3^). The main outcome of this study is the variation of the structure factors of confined CO_2_ phase, in comparison to bulk liquid, by the presence a shoulder on the right *Q* side of the main peak (*Q*=2.7 Å^−1^). The shoulder is mostly pronounced at 5 bar which corresponds to monolayer coverage. As a next step, the authors applied an experimental approach to eliminate the possible contribution of the adsorbed monolayer structure on one hand, while on the other to minimise the contribution of the cross correlation term (second term of Equation (6)). This was attempted by aiming to observe the “*true*” confinement effect, i. e. the molecular structure of CO_2_ only in the “*core*” of the pores (first term of Equation (6)). This approach was implemented by simply using for the corrections the experimental differential cross section (DSC) of the matrix loaded with CO_2_ at 5 bar (i. e. at monolayer formation), instead of the DSC of the empty matrix. This approach is valid under the assumptions that a) the correlations between the core fluid and the adsorbed film or the matrix are negligible and b) the molecular structure of monolayer and condensate are identical. Indeed, when this approach was applied the shoulder disappeared from all adsorption stages, strongly implying that it was originated from the monolayer structure and possibly the cross correlation (Figure 2). As a result, the confined CO_2_ at the “*core*” of the pore has similar properties with the bulk liquid.

The correlation functions have also suggested *liquid‐like* properties of the adsorbed CO_2_ molecules during all adsorption stages (Figure 3). In specific, the two intramolecular peaks are clearly visible for both liquid and sorbed fluid at about 1.16 Å and 2.32 Å, respectively. The intramolecular peaks arise from the C−O and O−O distances within the molecule (obviously, the O−O distance is twice the C−O distance). The intermolecular part of the correlation function presents two broad features centered at ~4 and ~8 Å, corresponding respectively to the first‐ and the second‐neighbour interactions. Additionally, a split of the first‐neighbour peak at three structures is observed for both the bulk liquid and the confined CO_2_, although certain differences can be observed (Figure 3). Despite the fact that sorbed CO_2_ molecules have liquid‐like properties during all adsorption stages, the subtle differences observed both at the structure factors and the differential correlation functions point to stronger orientational correlations inside the pores.^[46]^ These differences were attributed to either the interaction of the pore walls with the fluid (monolayer coverage) or the confinement of the fluid itself (when pores were saturated), combined with the large quadrupole moment of carbon dioxide. In another study, Stefanopoulos et al.^[47]^ have studied the structure of supercritical CO_2_ confined in the pores of MCM‐41 by sorption with *in situ* total neutron scattering. The results suggested that the structure of confined phase is similar to the bulk supercritical one, although some differences have also been observed. Again, these differences have been attributed to either the interactions between pore wall and supercritical CO_2_ molecules or the fluid confinement itself.

**Figure 4:**
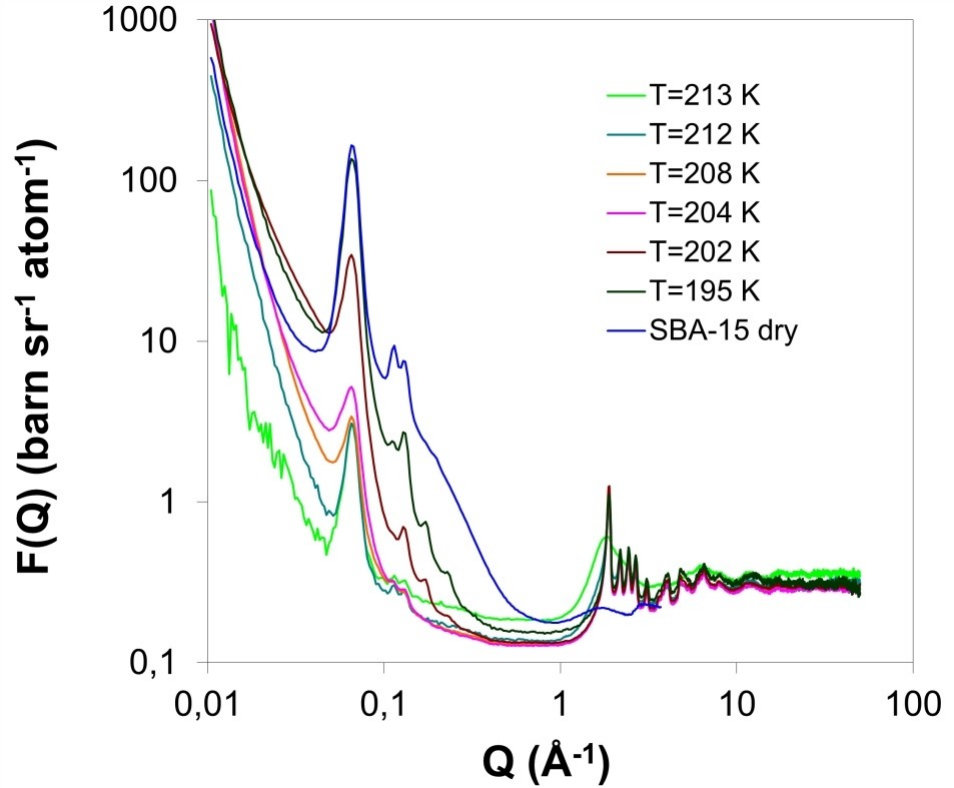
Diffraction profiles of SBA‐15 overfilled with CO_2_ (*T*=214 K) during freezing process. The pattern of dry SBA‐15 is also presented for comparison. Reproduced and modified from Ref. [48] with permission from APS.

Furthermore, Stefanopoulos et al.^[48]^ have also revealed the liquid‐like properties of CO_2_ confined in SBA‐15 by conducting *in situ* CO_2_ adsorption and neutron total scattering at 253 K and close to the bulk triple point (*T*_3_=216.55 K, *P*_3_=5.17 bar), respectively. SBA‐15 is another ordered mesoporous material with larger pores compared to MCM‐41. The experiment performed at NIMROD instrument[^38^, ^49^] (Figure 1), located at ISIS Neutron and Muon Source, UKRI Science and Technology Facilities Council (Oxfordshire, UK). NIMROD covers a wide *Q*‐range that bridges the gap between small‐ and wide‐angle neutron scattering (SANS‐WANS). From this point of view the adsorption mechanism, such as pore filling‐emptying, can also be monitored from the small‐angle regime of the scattering pattern. The most interesting outcome of this work was that upon freezing the saturated sample below the bulk triple point, CO_2_ molecules neither freeze nor remain liquid as expected, but escape from the pores (Figure 4). Furthermore, the process was fully reversible and during heating CO_2_ refilled the pores, however, with temperature hysteresis. This spontaneous depletion has been attributed to the fact that during cooling the vapour pressure of the bulk phase (solid CO_2_) drops below the relative pressure where desorption of the supercooled condensate should be expected. As a result, capillary forces cannot further retain the condensed phase and carbon dioxide molecules spontaneously escape from the pores to solidify externally.

Stefanopoulos et al.^[50]^ and Tampaxis et al.^[51]^ have also utilised *in situ* neutron total scattering and CO_2_ pore filling in another ordered mesoporous system (CMK‐3), an inverse replica of SBA‐15. Again, the experiment carried out at NIMROD instrument. The results of the saturated sample close to the bulk triple point, *T*_3_, revealed that confined CO_2_ is in a liquid‐like state, however, strongly densified; the calculated density was approximately 1.6 g/cm^3^, i. e. close to that of the bulk dry ice. This strong densification is explained on the basis of the overlapping solid wall potential fields in confined spaces coupled with strong fluid‐fluid attraction. As a result, these interactions force the confined fluid molecules to come close to each other by orienting their axis into preferred directions, in order to attain the highest possible packing density. In a similar way to SBA‐15, upon cooling below the bulk triple point, a depletion of pore‐confined CO_2_ takes place. In particular, the CO_2_ molecules leave the pore space to solidify externally. Again, depletion is fully reversible and, upon heating, CO_2_ progressively refills the large pores; the process is also associated with a temperature hysteresis. However, this process occurred only to secondary pores originating from incomplete ordering of nanorods.

Katsaros et al.^[52]^ and Steriotis et al.[^53^, ^54^] have investigated the structural properties of CO_2_ confined in a microporous carbon along an isotherm at 308 K (slightly above the critical temperature) and pressures up to 60 bar (below the critical pressure) by *in situ* adsorption and neutron diffraction.^[55]^ The evaluated structure factors and the correlation functions suggested that, although the pressures were well‐below the critical one, the confined phase was in a densified state with structure similar to a supercritical fluid or even bulk liquid CO_2_. It is noteworthy that the density of bulk CO_2_ at 308 K and 60 bar (gas phase) is 0.16 g/cm^3^; when confined within the micropores of the carbon sample, however, its density is increased almost 4 times to approximately 0.6 g/cm^3^. This densification has been explained by considering that inside the micropores the potential fields from the neighbouring pore walls overlap and produce very deep potential wells. As a result, there is an increase in the interaction energy between the fluid and the carbon matrix leading to enhanced adsorption, while the CO_2_ molecules are arranged within the micropores in very dense states with the highest molecular packing efficiency in order to compensate the lack of free volume. Furthermore, Grand Canonical Monte Carlo (GCMC) simulations revealed structural details for how the CO_2_ molecules are arranged within the slit graphitic pores of the microporous carbon.^[56]^ In another neutron scattering study, Bahadur et al.^[57]^ explored the structural properties of sorbed CO_2_ on an ultra‐microporous carbon specimen at room temperature. It was also concluded a strong densification of confined CO_2_ molecules within the carbon micropores and a density of ~1 g/cm^3^ was deduced. Again, Melnichenko and coworkers[^58^, ^59^] have studied the density of adsorbed CO_2_ in aerogels by neutron transmission and small‐angle neutron scattering (SANS). The results suggested that a porous aerogel matrix works to create an adsorbed phase with liquid‐like fluid densities reaching ∼1.1 g/cm^3^ and ∼0.8 g/cm^3^ (compared to the critical density, *ρ_c_* =0.468 g/cm^3^) corresponding to temperatures, *T*=35 °C and *T*=80 °C, respectively. Despite the fact that the density of the adsorbed CO_2_ decreases with temperature, the dense adsorbed phase is still present in the aerogel at temperatures far exceeding the critical one. They concluded that the adsorption of supercritical CO_2_ in aerogels is governed predominantly by long‐range van der Waals interactions between the silica skeleton of the aerogels and fluid molecules.

Apart from carbon dioxide, there are also neutron scattering studies related to the structural properties of other fluids such as water confined in porous materials. Bellissent‐Funel et al.^[60]^ investigated the structure of deuterated water confined in the interconnected pores of Vycor porous glass, as a function of temperature, by neutron scattering. One important finding of this study was that the hydration level plays an important role to the structural changes upon cooling down. For instance, the amount of supercooling of water in partially hydrated Vycor porous glass is much more compared to the fully hydrated sample. The observed phase of ice in the fully hydrated Vycor is the cubic ice, which appears at about −18 °C. Moreover, traces of liquid water are still present at −41.5 °C in both fully and partially hydrated samples. Soper and coworkers[^61^, ^62^, ^63^] have used neutron scattering in conjunction with *isotopic substitution*[^64^, ^65^, ^66^, ^67^] of deuterium (D) with hydrogen (H) to study the structure of both bulk and water confined in Vycor porous glass. In the case of confined water they also combined the neutron scattering data with the Empirical Potential Structure Refinement (EPSR) developed by Soper.[^68^, ^69^, ^70^] They concluded that the structure of water confined in Vycor is strongly perturbed relative to the bulk. In particular, they observed both a significant decrease in the first shell water oxygen‐oxygen co‐ordination number and a decrease in the number of hydrogen bonds per water molecule from 3.6 in bulk water to 2.2, in confinement. The significance of the excluded volume effects is also discussed. In addition, Soper^[71]^ combined total neutron scattering measurements (with *isotopic substitution*) and atomistic simulations (EPSR) to investigate the structure of water confined in the cylindrical pores of MCM‐41. He observed that the calculated average local density of water in the pores was about 20 % lower than bulk water density, although the density in the core region was still below, but closer to the bulk density. The most striking result was that the pressure of the water inside the pore would have to be negative to the value of around −100 MPa (−1 kbar).

Furthermore, a series of neutron scattering experiments were carried out for the structural characterisation of water and ice in mesoporous SBA‐15 according to the degree of pore feeling at D_2_0 Diffractometer, Institut Laue Langevin (ILL) Grenoble (France). In the case of the SBA‐15 sample “*over‐filled*” with water, it was observed that hexagonal ice is initially formed on the outside of the silica grains, followed by the nucleation of cubic ice inside the pores. Moreover, below the pore freezing temperature, the results indicated a reversible conversion of defective ice to ordered ice crystals.^[72]^ When the sample was “*almost‐filled*” the primary nucleation event at 258 K resulted in a defective form of ice‐I with predominantly cubic ice features. For temperatures below the main nucleation event, the results indicated the formation of an interfacial layer of disordered water/ice that varies with temperature and is reversible.^[73]^ Further analysis of the results suggested that the phase relationship of ices in confined geometry is more complex than has been previously realized. More specifically, the authors claim the co‐existence of several different structural phases of ice although it is not possible to locate with precision these inhomogeneities within the pore volume.^[74]^ Finally when the SBA‐15 sample was “*partially filled*” with water, the ice formed in the pores had characteristics that differed markedly from those seen in the previous measurements.^[75]^ For instance, a significant amount of hexagonal ice was observed. In general, the outcome of all the above studies on water confinement in the pore network of SBA‐15 in various levels of pore filling suggested the complexity of the nucleation process and the different forms of ice created.

The sensitivity of neutrons to light elements has been utilised by Soper and Bowron[^76^, ^77^] to study the structure of simple gases (N_2_, O_2_, D_2_, and CD_4_), upon adsorption on ordered mesoporous silica MCM‐41. Adsorption was achieved by immersing MCM‐41 in a bath of the relevant gas, keeping the gas pressure constant (1 bar) and lowering the temperature in steps toward the corresponding bulk liquid boiling point. The neutron scattering measurements combined with atomistic computer modeling to interpret the experimental data. During adsorption process all four gases show closely analogous behaviours, with an initial formation of liquid‐like layers on the inside surface of the pores, followed by a relatively sharp capillary condensation when the pores become filled with dense fluid. Noteworthy, the estimated density of all the sorbed liquids was either slightly below that of the bulk saturated liquid at the same temperature or, in the cases of CD_4_ and D_2_, very much below the bulk liquid density. Furthermore, the capillary condensation is signaled by a sharp decrease in the intensity of (100) Bragg diffraction peak arising from the hexagonal array of the cylindrical pores of MCM‐41. The condensation transition is accompanied by a small but significant expansion of the lattice and considerable additional diffuse scattering due to the existence of heterogeneities along the pore axis. Conclusively, such heterogeneities could arise either from vapour bubbles in the adsorbed fluid or from the inner pore surface having a degree of roughness.

Neutron scattering has also a unique power to investigate catalytic phenomena. Yu et al.^[78]^ have published a comprehensive overview of recent advances in neutron scattering investigations of heterogeneous catalysis, focusing on surface adsorbates, reaction mechanisms and catalyst structural changes elucidated by neutron spectroscopy, diffraction and other neutron techniques. In addition, Parker^[79]^ reviews the role of neutron scattering methods related to “*Net Zero*” and catalysis. The “*Net Zero*” is aiming to achieve equality between the amount of produced greenhouse gas emissions and the amount removed from the atmosphere. The authors discuss how neutron scattering can be utilised in order to study reactions and processes that are directly relevant to achieving “*Net Zero”* such as CO_2_ capture and exploitation. In case of neutron total scattering, the structural transformations of catalytic materials at the nanoscale can be monitored in real time. For instance, an interesting application was the study of the platinum catalysed hydrogenation of aromatic molecules in MCM‐41.[^80^, ^81^] The results gave a strong evidence that neutrons can probe the reaction within the pore of the catalyst. The experiments carried out at NIMROD instrument which is able to provide kinetic information of reaction processes over microscopic, mesoscopic and macroscopic length scales, simultaneously. In addition, the structural details of the pore‐confined aromatic liquids can also be refined. In the first study, Youngs et al.^[80]^ introduced deuterated benzene (benzene‐*d*_6_) followed by exposure to D_2_, which results in the catalysed hydrogenation of the aromatic to cyclohexane‐*d*_12_. The evolution of the structure factor as a function of time illustrates the structural changes during the liquid phase reduction of benzene. Moreover, by taking slices at particular *Q* values with respect to time, the kinetics corresponding to different length scales in the system have been deduced and the rate of the reaction constants have also been extracted. The values of time constants suggest that the overall process is likely to be limited by liquid diffusion, as this is the slowest rate observed, while the overall reaction rate is governed by the hydrogenation process rather than the dissociation of D_2_. In the second study, Falkowska et al.^[81]^ introduced hydrogenation (D_2_) of deuterated toluene (toluene‐*d*_8_) on MCM‐41, which results to methylcyclohexane‐*d*_14_. Again, three different rate constants were identified, from which the slowest one was that describing liquid rearrangement in the pores due to the product formation. Noteworthy, a comparison between toluene‐*h*_8_ and toluene‐*d*_8_ showed that there was no kinetic isotopic effect present in either of the steps of heterogeneous process observed. This finding strongly suggests that *isotopic substitution* can be employed for the exploration of catalytic phenomena. Apart from obtaining simultaneous kinetic information associated with chemical reaction and mass transport within the pore of a catalyst, neutron total scattering in conjunction with atomistic simulation can further reveal the structural details from the confined liquids. Moreover, neutron experimental results from the bulk liquids are also necessary for comparison reasons.[^82^, ^83^, ^84^] In the case of benzene,^[85]^ the nanoscale confinement has a major effect on the spatial and orientational correlations between the molecules, when compared with the structure of the bulk liquid. These correlations were most pronounced when the molecules are in parallel configurations, suggesting differences in chemical reactivity between the confined and bulk liquids.

**Figure 5:**
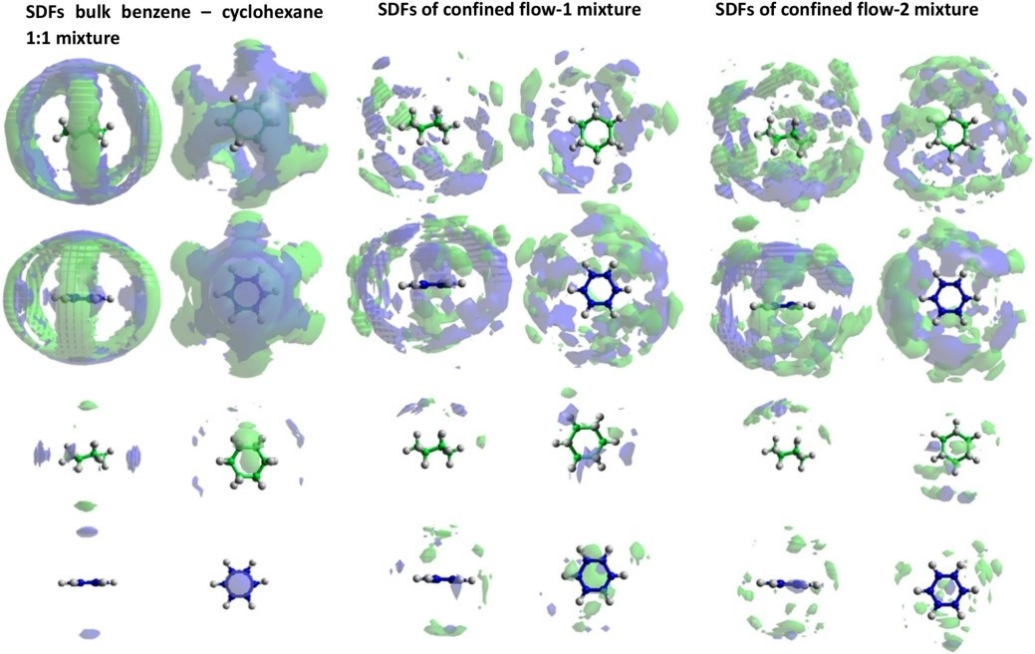
The spatial density functions of parallel molecules (z‐axis *θ*=0±10°) within the first coordination shells at distances r>4.95 Å (top double row) r<4.95 Å (bottom double row) of the a) 1 : 1 benzene to cyclohexane bulk mixture, b the flow 1 confined system and c the flow 2 confined system. Cyclohexane molecule and cyclohexane corresponding spatial density are represented in green, whereas benzene molecules and benzene corresponding spatial density are represented in blue. Reproduced from Ref. [87] with permission from Springer.

Furthermore, *in situ* neutron experiments with hydrogenation of deuterated toluene combined with NMR spectroscopy can provide valuable information about the fluid composition. The use of NMR can be proved to be crucial, as otherwise it is not possible to assign the structural changes to the formation of intermediate products, as these products have often very similar structure and they might be indistinguishable by the neutrons on their own.^[86]^ By combining neutron experiment in conjunction with NMR, mixtures of cyclohexane and benzene have been investigated in both the bulk liquid phase and when confined in MCM‐41.^[87]^ The experimental results combined with EPSR simulations suggested, that upon confinement of the hydrocarbon mixtures, a stronger parallel orientational preference of unlike molecular dimers at short distances. Furthermore at longer first coordination shell distances, the spatial organisation of benzene molecules within the mixture has also showed variations upon confinement. More specifically, parallel oriented molecules, rather than perpendicular, found directly above and below the central rings (Figure 5). The dynamics of bulk benzene and benzene confined in the pores of Pt/MCM‐41 have also been probed by quasi‐elastic neutron scattering (QENS).^[88]^ The results combined with molecular dynamics (MD) showed that the mobility of benzene decreases upon confinement as confirmed by the decreased diffusion coefficients. As a general remark, the combined QENS‐MD studies proved to be an essential tool in quantifying the dynamics and studying the qualitative behaviour of the system. Analysis of the dynamics revealed an intriguing combination of unhindered translational and jump diffusion.

As a final but important component of this section, metal‐organic frameworks (MOFs) have recently attracted a lot of interest in various fields, such as gas storage (including CO_2_) and separation, sensor, catalysis, energy storage and biological engineering.[^89^, ^90^, ^91^, ^92^] MOFs are crystalline porous materials composed of metal‐based building units coordinated to organic bridging ligands to form a three‐dimensional network with uniform pore system including channels and cages. Neutron diffraction and scattering methods offer unique possibilities for determining key features of their complex structural characteristics. Moreover, the increasing demands for further understanding their crystallisation and crystal growth mechanisms towards a more controllable and rational design, results in the development of *in situ* diffraction and combined‐diffraction techniques. Furthermore, when performing *in situ* neutron diffraction and gas loading on MOFs, valuable information can be gained both about the precise locations of the guest gas molecules within the pore cavities and their interactions with the crystalline framework. Finally, *in situ* neutron diffraction is an appropriate technique to explore the properties of nanoconfined adsorbate phases and to understand how they might differ from the respective bulk properties.[^93^, ^94^, ^95^, ^96^, ^97^]

## 3 Small‐Angle Neutron Scattering (SANS) from Confined Fluids

Complementary to neutron total scattering that measures interatomic distances, SANS probes the structures at larger length scales, varying from 1 nm to a few hundred nanometers while USANS probes lengths up to a few micrometers (about 10 μm).[^98^, ^99^, ^100^] (U)SANS is an essential tool for the structural characterisation of porous materials by providing information about the geometry and topology of the pores, the interface texture (smooth or rough), the total porosity, the pore size distribution and the specific surface area. Moreover, when combined with adsorption, essential information can also be gained about the structural properties and the phase behaviour of the pore‐confined fluid.^[101]^ For an in‐depth understanding of the SANS technique to study the phase behaviour of fluids confined in porous solids with applications to energy storage and environmental science I would suggest the book by Melnichenko.^[14]^ According to SANS theory, the scattered intensity of a two‐phase system (for instance, a porous solid consisting of the solid matrix and the pore space) can be expressed as:^[102]^

(7) $$I\left(Q\right)=N_{p}V_{p}^{2}{\left(\rho_{p}-\rho_{s}\right)}^{2}P\left(Q\right)S\left(Q\right)$$

where *N_p_* is the number density of the scattering particles (pores), *V_p_* is the particle (pore) volume, *ρ_p_* and *ρ_s_* are the scattering length densities (SLDs) of the pores and the solid matrix, respectively. It can be seen that the scattered intensity is proportional to the square of the *contrast term* defined as the difference between the SLD of the pore content (*ρ_p_*) and the SLD of the solid matrix (*ρ_s_*), respectively. Obviously for a porous solid with empty (filled with air or evacuated) pores, *ρ_p_* =0;^[103]^ alternatively, when the pores are filled with a fluid, *ρ_p_* = *ρ_f_*, where *ρ_f_* is the SLD of the fluid.

*P(Q)* is a dimensionless function known as the *form factor* depending on the size and the shape of the scatterers, such as pores. In general, there are analytical expressions for the form factor of particles with simple shapes such as sphere, disk and thin rod.^[104]^ Additionally, the form factor can be directly measured when the number density of the scattering objects is small (such as dilute systems) and, therefore, the distance between the scatterers is much larger than their sizes.

*S(Q)* is another dimensionless factor, the *structure factor* arising from interference in the scattering from particles (pores) in close separation. In general, the structure factor provides information about spatial arrangements of the scattering objects related to their correlations in their position. In case of dilute scattering objects, only the form factor is taken into account because *S(Q)*=1.

One of the important parameters of the SANS technique is the *scattering length density* (SLD). The neutron SLD, *ρ*, of a molecule is a measure of the average scattering power of neutrons by the atoms within that molecule. In principle, it quantifies the ability of neutrons to interact with the atomic nuclei in the molecule and it is defined as:

(8) $$\rho =\sum_{i=1}^{n}\frac{b_{i}}{V_{m}}=\sum_{i=1}^{n}\frac{b_{i}dN_{A}}{M_{w}}$$

where *b* is the bound coherent scattering length and the summation is over every atom (*i*) in the molecule with a molecular volume *V_m_*; *M_w_* is the molecular weight, *d* is the physical density and *N_A_* is the Avogadro number. In case of multicomponent samples such as geological systems, the average SLD, *ρ*_multi_, can be calculated as a volume average over all compounds:

(9) $$\rho_{\mathrm{multi}}=\frac{1}{100}\sum_{i=1}^{n}\phi_{i}\rho_{i}$$

where *n* is the total number of compounds, *ϕ_i_* is the volume concentration of the *i*‐th compound (in percent) and *ρ_i_* is the SLD of the *i*‐th compound. For instance, a shale sample may be consisted of components like quartz (SiO_2_), carbonates (CaCO_3_), pyrite (FeSO_2_), zircon (ZrSiO_4_), etc.

One of the benefits of the SANS technique is the utilisation of *contrast matching*. The fact that H and D have scattering lengths and, thus SLDs, of opposite sign^[105]^ means that by filling the pores using an appropriate mixture of hydrogenous and deuterated liquid solvents (such as H_2_O/D_2_O) the SLD of the porous solid (*ρ_s_*) becomes equal to that of the solvent mixture (*ρ_f_*) and *contrast matching* is attained; as a result, the scattering signal is eliminated because the contrast term becomes zero (see Equation (7), when *ρ_s_* =*ρ_p_* =*ρ_f_*). Apparently, the absence of a SANS signal is an evidence that the porous material contains only open (accessible) pores saturated by the liquid mixture. On the other hand, any observed residual signal will yield information about the presence of closed (inaccessible) pores, which the liquid cannot penetrate; this residual scattering arises from the contrast between the SLDs of the matrix and the unfilled pores respectively. Obviously, these pores do not belong to the interconnected porous channels, which have access to the external surface. Consequently, *contrast matching* SANS is a powerful tool for a direct investigation and evaluation of the closed porosity in porous media. *Contrast matching* SANS has been extensively used to evaluate the fraction of inaccessible pores using liquid mixtures of protonated and deuterated solvents in coals,[^13^, ^106^, ^107^, ^108^] coal chars[^109^, ^110^] and doped graphites.^[15]^

**Figure 6:**
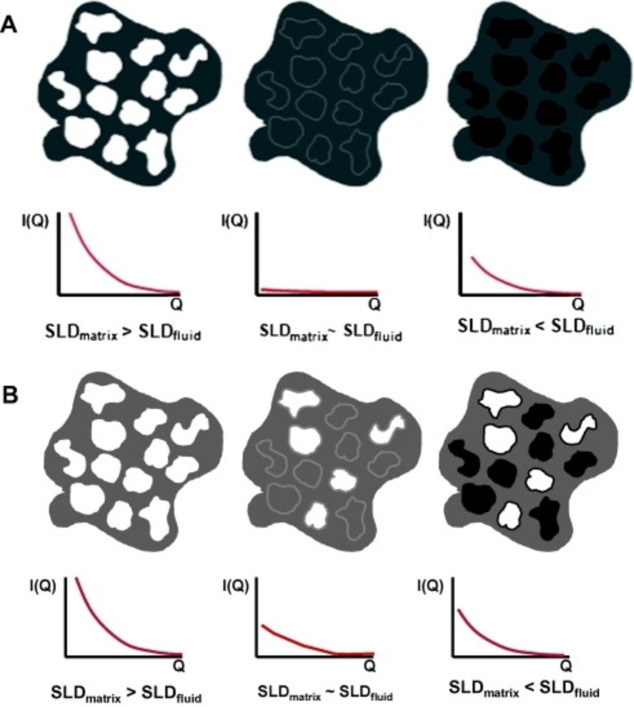
Qualitative presentation of *contrast matching* experiments with fluid saturated porous systems. (A) All pores are accessible to fluid molecules; (B) Pores are partially accessible to fluid molecules. In the latter case, the residual scattering at the *zero average contrast* condition can be used to quantify the volume fraction of accessible pores as a function of pore sizes, as explained in the text. Reproduced from Ref. [111] with permission from Elsevier.

However, there are some cases, like coal, where many of the functional groups can exchange hydrogen with water on time scales varying from seconds to weeks. As a result, the *isotope exchange* may alter the H_2_O/D_2_O ratio in pores and significantly shift the local *contrast matching* condition. Melnichenko and coworkers^[111]^ suggested an alternative method of achieving *contrast matching* in porous media by using non‐adsorbing or weakly adsorbing supercritical fluids or gases such as carbon dioxide, CO_2_ or deuterated methane, CD_4_ (it is preferred instead of CH_4_, for minimising the incoherent background scattering from hydrogen). In a similar way with the liquid mixtures, by measuring the neutron scattering patterns as a function of fluid pressure, when the *contrast‐matched pressure* (or the *zero average contrast*, ZAC) is reached, any residual scattering signal simply indicates the presence of pores inaccessible to CO_2_ or CD_4_ (Figure 6). The volume fraction of accessible pores, *C*_ac_, can be then calculated at any values of the scattering vector, *Q*, from the ratio of the scattered intensities at ZAC pressure and at zero pressure (under vacuum, dry sample, empty pores), according to the simple expression:

(10) $$\frac{I\left(P_{\mathrm{ZAC}}\right)}{I\left(P_{0}\right)}=1-C_{\mathrm{ac}}$$

**Figure 7:**
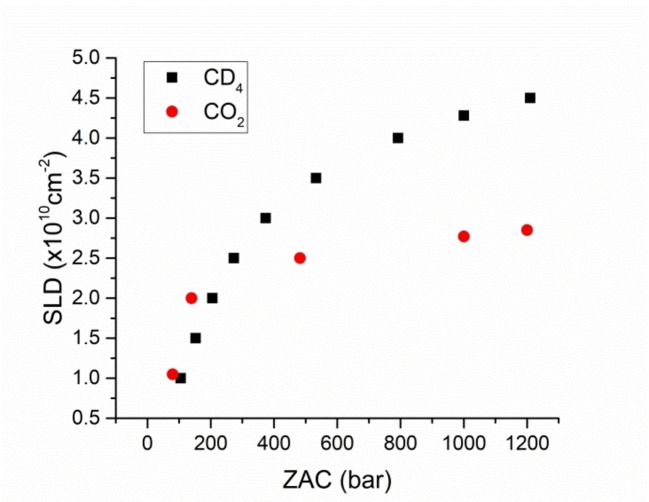
SLD values as a function of *Zero Average Contrast* (ZAC) for supercritical CD_4_ (25 °C) and supercritical CO_2_ (35 °C), respectively. The SLD values at 1 kbar are 4.28x10^10^ cm^−2^ for CD_4_ and 2.77×10^10^ cm^−2^ for CO_2_, respectively.

From the above equation the ratio of the volume of the accessible pores to the total pore volume can be calculated as a function of the scattering vector or the pore size (note that for the pore size, *D*, of the rocks there is an approximate relationship which is an analogue of Bragg's law: *D*=5/*Q* ^[112]^). It is noteworthy that an important benefit of utilising gases or supercritical fluids for *contrast matching* is their excellent penetration into porous network because of their much lower viscosity compared to corresponding liquids. Figure 7 presents the SLD values of supercritical CD_4_ and CO_2_ as a function of their *contrast matching* pressure (ZAC). According to Equation (8) the SLDs of CD_4_ and CO_2_ vary with their physical densities as:^[111]^

(11a) $$\rho_{{\mathrm{CD}}_{4}}=\left(10\cdot d_{{\mathrm{CD}}_{4}}\right)10^{10}\;{\mathrm{cm}}^{-2}$$

(11b) $$\rho_{{\mathrm{CO}}_{2}}=\left(2.49\cdot d_{{\mathrm{CO}}_{2}}\right)10^{10}\;{\mathrm{cm}}^{-2}$$

It is well known that CO_2_ is subcritical gas at room temperature.^[55]^ Furthermore, subcritical bulk CO_2_ condenses into a liquid at 57 bar (at room temperature). As a result, its SLD can cover only a limited range as a function of pressure and, thus, high‐pressure supercritical CO_2_ is required to attain the *contrast matching* condition for a variety of porous materials. For instance, the SLD of CO_2_ at 35 °C and 1 kbar is 2.77x10^10^ cm^−2^ (Figure 7).^[113]^ This means that even supercritical CO_2_ is not sufficient to achieve *contrast matching* for a wide range of sedimentary rocks such as shale, carbonates, sandstones and mudstones because their SLD is greater than 3x10^10^ cm^−2^. On the other hand, the fact that CD_4_ is supercritical at room temperature means that it can be the fluid of choice in order to attain a wide range of SLDs. It is worth mentioning that at room temperature (25 °C) and at pressure 1 kbar the SLD for CD_4_ is 4.28x10^10^ cm^−2^ (Figure 7). This value is sufficient enough to *contrast‐match* a wide class of porous media.

**Figure 8:**
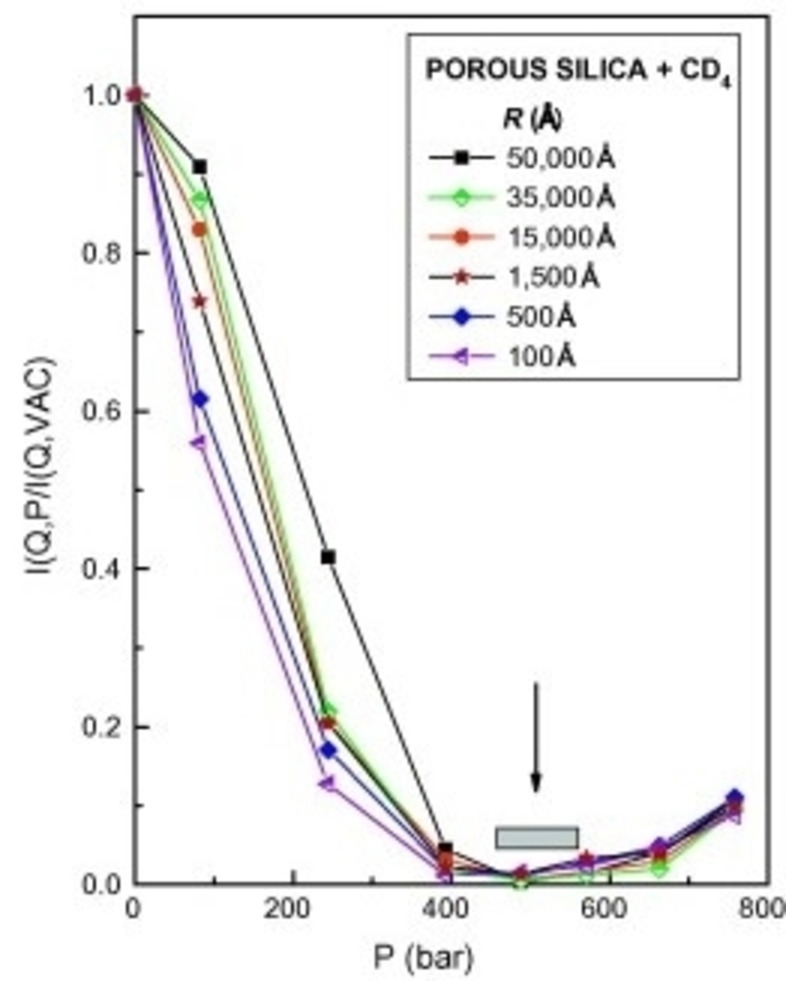
Variation of the normalised cross section as a function of pressure of D‐methane for porous fractal silica in pores of different sizes *R*. The arrow shows calculated pressure *P*_CM_=514 bar at which *contrast matching* point should be reached. Pore sizes estimated using relation *R*~2.5/*Q*. Reproduced and modified from Ref. [111] with permission from Elsevier.

**Figure 9:**
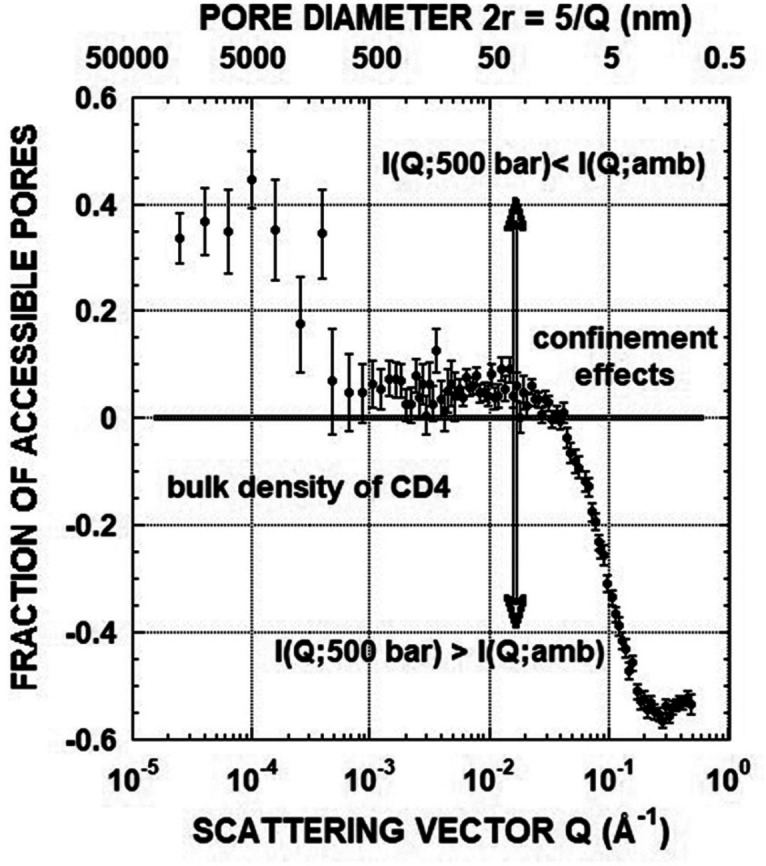
Plot of the apparent fraction of accessible pores, *F_a_*, calculated for the sample Marcellus_7084 according to Eq. A1. The region of pore sizes containing gas with the bulk pressure and density values is delineated from the nanopores which host gas of higher density (increasing as the pore size decreases due to the influence of confinement effects) by the vertical double arrow. The borderline is located at *Q*~2×10^−2^ Å^−1^, which corresponds to the nanopore diameter of ~25 nm. Pore diameters, calculated for every full decade of *Q* values, are displayed above the top horizontal axis. Negative values of *F_a_* are an artefact discussed in the text. Reproduced from Ref. [114] with permission from Elsevier.

If we assume a binary system (porous material) with structural homogeneity, the ratio $\frac{I\left(P\right)}{I\left(P_{0}\right)}$ can be measured as a function of the pressurised fluid for all values of *Q* (or pore sizes). In the absence of closed pores, the scattered intensity will show a monotonic decrease as the pressure approaches the calculated *contrast matching* condition (or ZAC pressure), followed by a monotonic increase (Figure 8). The nearly zero value of the intensity around ZAC pressure for all values of *Q* indicates the absence of closed pores and negligible SLD fluctuations. This is the case of a porous silica sample upon injection of pressurised CD_4_.^[111]^ The *contrast matching* pressure was calculated to be 514 bar using the physical density of amorphous silica (*d*=1.8 g/cm^3^). As shown in Figure 8, the intensity minimum (shown by the arrow) is identical with the ZAC pressure. As a next step, Radliński and coworkers,^[114]^ showed that the confinement effect on the phase behaviour of fluids can be also revealed for particular pore sizes, when the experimental value of the fraction of accessible pores, *C*_ac_ (or *F_a_*, according to their notation), becomes negative (Equation (10)). This behaviour is attributed to the increased gas density in confinement, which violates the *contrast matching* condition (Figure 9).

The evaluation of accessible and inaccessible porosity using *contrast matching* by exposing the porous media to high‐pressure supercritical fluids might affect their pore morphology due to mechanical distortions.^[115]^ An alternative methodology has been developed by Bahadur et al.,^[116]^ without the requirement of reaching the *zero average contrast*, ZAC. More specifically, by taking the ratio of intensities at various fluid pressures at specific values of *Q* (or pore size) and, by knowing the ratio of their contrasts, the volume fractions of open and closed pores can be calculated:

(12) $$\frac{I\left(P\right)}{I\left(P_{0}\right)}=S\left(P\right)C_{\mathrm{ac}}\;+\;C_{\mathrm{in}}$$

where *I*(*P*) and *I*(*P*_0_) are the scattered intensities at fluid pressure *P* and at zero fluid pressure (or under vacuum, where the pores are empty), *C*_ac_ and *C*_in_ are the volume fractions of accessible and inaccessible pores respectively,

(13) $$S\left(P\right)={\left[1-\frac{\rho_{f}\left(P\right)}{\rho_{m}}\right]}^{2}$$

*ρ*_f_(*P*) and *ρ*_m_ are the neutron scattering length densities (SLDs) of the fluid at a given pressure *P* and the solid matrix respectively. The gradient and the intercept of the line derived from Equation (12) give the volume fractions of accessible and inaccessible pores at each *Q* value that corresponds to a definite pore size or pore radius (according to the empirical relation *D*=5/*Q*). As a result, the values of *C*_ac_ or *C*_in_ as a function of pore size can be deduced. Noteworthy, this method can be applied by assuming: (i) no significant confinement of the fluid in the pores and (ii) similar pore morphology (size and shape) of the accessible and inaccessible pores. As it will be discussed in the next section, the pore accessibility of CO_2_ in various sedimentary rocks is one of the key parameters for sequestration and enhanced oil recovery.

Combination of gas adsorption and SANS (or *contrast matching* SANS) in porous materials has also been proved to be an essential tool for both highlighting the structural details of the porous matrix and the phase behaviour of the adsorbed fluid. Moreover, the technique provides the unique possibility to elucidate both the adsorption mechanism and the kinetics of adsorption. For instance, this can be achieved in mesoporous materials by monitoring the processes involved such as micropore filling, formation and growing of adsorbed mono‐ and multilayers and capillary condensation in mesopores.[^117^, ^118^, ^119^, ^120^, ^121^, ^122^, ^123^, ^124^, ^125^, ^126^, ^127^, ^128^, ^129^, ^130^, ^131^, ^132^, ^133^]

As a final part of this section, we will highlight SANS applications to ionic liquids (ILs) confined in porous materials. ILs represent an important class of fluids consisting entirely of ions and being liquid below 100 °C. They possess unique properties such as good electrolytic and solvation properties, excellent electrochemical and thermal stability, non‐volatility and non‐flammability. As such, ILs have improved performances in many areas such as electrochemistry, analytical chemistry, gas separation technologies, synthesis and heterogeneous catalysis, heat transport and conversion.[^134^, ^135^] Nowadays, ILs are considered as alternative solvents for addressing the high energy demand of CO_2_ capture. More specifically, ILs can bring step changes in the efficiency of all currently available CO_2_ capture technologies, being directly applied as liquid absorbents, or used as adsorbent and membrane modifiers that enhance the CO_2_ selectivity.^[136]^ As a result, an understanding of the confinement of ILs in nanoscale geometries, such as pores, is of fundamental interest.^[137]^ From this point of view, application of neutron scattering techniques provides essential information about the interaction between ILs and solid surfaces as well as the influence of their confinement on their structure and morphology.^[14]^ For instance, the IL accessibility to the pores of ordered mesoporous silica materials (MCM‐41 and SBA‐15) can be investigated in a unique way by SANS and *contrast matching* SANS.[^138^, ^139^, ^140^] The reason is that neutron scattering techniques have the advantage over the conventional gas adsorption methods that can provide information for the bulk of the pores; as previously discussed, this means that neutrons can also “*see*” the pores that are inaccessible to gas molecules, such as pores that are blocked due to deposition of the ionic liquid near the pore aperture. In this case the negligible uptake from the sorption isotherm would erroneously suggest that the pores are totally filled with IL.

## 4 Applications to CO_2_ Sequestration and Enhanced Oil and Gas Recovery

Neutron scattering has recently gained significant attention as a powerful tool to reveal key properties of sedimentary rocks such as their structure, their mechanical properties, as well as the properties of their complex pore network. Neutron scattering can also provide essential information about the structure and dynamics of confined reservoir fluids in the rock matrix.[^14^, ^141^, ^142^, ^143^, ^144^, ^145^, ^146^, ^147^, ^148^, ^149^] *Contrast matching* SANS is a unique technique to evaluate the accessibility of pores to various fluids in order to assess the pore connectivity of sedimentary rocks. Differentiating accessible (open) and inaccessible (closed) porosity is very important for CO_2_ sequestration and enhanced oil and gas recovery (EOR, EGR). The reason is that closed pores could play a crucial role in EOR‐EGR because they might also contain hydrocarbons; as a result, the efficiency of the CO_2_‐EOR (or CO_2_‐EGR) process would also depend on the extent to which the pores can be accessed. Furthermore, an amount of the injected CO_2_ could be safely stored in the reservoir, resulting in a decrease of the greenhouse emissions.

Coals are classified in “*ranks*” referring to the steps of “*coalification*”, which is a slow natural process which coal undergoes after deposition of organic material. Coal is classified into four ranks: anthracite, bituminous, subbituminous and lignite. The ranking depends on both the types and amounts of carbon the coal contains, as well as on the amount of heat energy the coal can produce. Briefly, anthracite (or hard coal) is the highest rank containing a high percentage of fixed carbon content (>91 wt %) and a low percentage of volatile matter. Bituminous coal is a middle rank coal (with 80–91 wt % carbon content), which is used in electricity generation and steel making in the USA. Noteworthy, both bituminous coals and anthracites consist of moderately and highly ordered graphitic layers. Low‐rank coals such as lignites and subbituminous coals are mainly used for electricity generation. They have carbon content <80 wt %, higher porosity than bituminous coals and they consist of randomly oriented layers.^[14]^ All types of coal have a heterogeneous, anisotropic and multi‐scale network of pores including all types of pores, micropores (<2 nm), mesopores (2–50 nm) and macropores (>50 nm). To this end, (U)SANS techniques combined with traditional porosimetry methods are essential to reveal the complex microstructure of coal.^[150]^ Coalbed methane (CBM) is a vital natural gas resource with a relatively low risk of development and its utilisation has grown rapidly during the last few decades, especially in the USA. Understanding the coal pore structure is a key parameter for the optimisation of CBM recovery, as they are closely related to both the gas content in coal and the coal permeability. Significantly, deep seam reservoirs have also been considered promising candidates for permanently sequestration of anthropogenic CO_2_ emissions, mainly due to their high internal surface area. Additionally, supercritical CO_2_ can be utilised to displace CH_4_, in order to achieve both enhanced coalbed methane (CO_2_‐ECBM) and geological sequestration, respectively.^[151]^ The synergistic effect of methane recovery and CO_2_ sequestration increases economic benefits, while reduces sequestration costs and helps to decrease CO_2_ emissions.

Shale is the most abundant sedimentary rock and the most common rock for hydrocarbons (oil and natural gas). Shale is a fine‐grained sedimentary rock, which is composed of silt‐sized grains of minerals (such as quartz, feldspar, calcite, dolomite and pyrite) and flake‐shaped grains of clay minerals (dominated by illite). Organic matter (OM), is an important component of shale in the forms of kerogen, bitumen and pyrobitumen/char.^[152]^ The concentration of organic matter (OM) is expressed as the percentage of total organic carbon (TOC) in the rock. In general, shales with TOC contents >1 wt % (preferably in the order of 2 wt % or even higher) are assumed to have enough hydrocarbon storage capacity and the potential for their extraction.^[145]^ As a source rock, shale has low porosity, ultra‐low permeability and its heterogeneous and anisotropic pore structure is even more complex in comparison to coal. As a result, pore structure characterisation of shale is very important but difficult task because a major fraction of the total pore volume is distributed in nanopores associated with OM and various inorganic minerals (mainly clay). Noteworthy, kerogen nanopores are considered to be the major host for oil and gas, although minerals (especially clay) are also associated with the presence of oil.^[145]^ Again, conventional porosimetry methods combined with (U)SANS techniques are necessary to quantitatively characterise the complex and multi‐scale pore structure in shale reservoirs.[^144^, ^145^] In general, horizontal drilling and induced artificial fractures by hydraulic fracturing (fracking) are used to produce oil and gas from unconventional shale reservoirs.^[153]^ Alternatively, unconventional shale reservoirs can also be attractive for CO_2_ tertiary enhanced oil recovery (CO_2_‐EOR)[^154^, ^155^, ^156^] and potential candidates for geologic CO_2_ sequestration.^[157]^ In case of natural gas, CO_2_‐enhanced gas recovery (CO_2_‐EGR) is also considered a promising technology to enhance shale gas recovery by injecting supercritical CO_2_ instead of water as the stimulation fluid. Additionally, CO_2_‐EGR and CO_2_ sequestration in depleted shale gas reservoirs could also be a cost‐effective strategy to permanently store CO_2_ in underground shale formations.^[158]^

Understanding the confinement effect of greenhouse gases, such as CO_2_ and CH_4_, in sedimentary rocks is a key factor in order to evaluate both the potential for geological CO_2_ sequestration and the hydrocarbon production efficiency. Neutron total scattering provides a unique opportunity to reveal the structural properties and the phase behaviour of confined geo‐fluids under various thermodynamic states. In addition, (U)SANS is also a unique tool to monitor the confined gas phase behaviour in varying‐sized pores at *P*‐*T* conditions of the reservoir. Furthermore, pore connectivity of sedimentary rocks is also a controlling factor for hydrocarbon storage and gas migration. *Contrast matching* (U)SANS has been proved to be an essential tool to investigate the accessibility of pores to gases in geomaterials.

As discussed in the previous section, Melnichenko and coworkers were the first to propose a methodology to determine the volume fraction of accessible pores in natural porous systems by utilising *contrast matching* SANS. This can be achieved by *in situ* injecting greenhouse gases (such as CO_2_ and CH_4_) at the *zero average contrast* (ZAC) pressure (or the *contrast‐matched* pressure). Melnichenko et al.^[111]^ carried out *contrast matching* SANS and USANS measurements to study the interconnectivity of pores in three different coal samples (one from the Illinois Basin in the USA and two others from the Bowen Basin, Queensland, Australia) by *in situ* injecting supercritical carbon dioxide and d‐methane. Their results provided first experimental evidence of the existence of closed pores in coal, which were inaccessible to the molecules of supercritical CO_2_ and CD_4_. In another study, Zhang et al.^[159]^ proposed a theoretical model to quantify pore accessibility for methane in two coal samples (one of sub‐bituminous rank and the other of anthracite rank) based on the scattering intensities at both vacuum and ZAC conditions. Their results demonstrated that pores smaller than 40 nm in radius are less accessible for anthracite than sub‐bituminous coal. However, for larger pore radii (>40 nm), the pore accessibility of anthracite coal becomes larger compared to that of sub‐bituminous one. Noteworthy, pore accessibility and pore radius follow a power‐law relationship for both coals. He et al.^[160]^ have also used SANS/USANS techniques to determine the inaccessible pore volume to CO_2_ and CD_4_ in four bituminous (middle‐rank) coals. Their results suggested condensation and strong densification of both fluids in pores with radius smaller than ∼36 Å. They also concluded that the evaluated inaccessibility to both fluids is related to physical or chemical properties of the coal matrix. Furthermore, Radlinski et al.^[161]^ attempted to calculate the density of sorbed CO_2_ in two coal samples (Seelyville and Baralaba) by carrying out *in situ* (U)SANS and SAXS measurements in a pressure range up to 50 bar and at temperatures 16 °C (Seelyville) and 35 °C (Baralaba), respectively. They found that the density of CO_2_ confined in coal depends on the pore size and can exceed the density of bulk CO_2_ at the same thermodynamic conditions by a factor of 2 to 5. They also observed that CO_2_ firstly adsorbed in the smallest micropores and then in the larger ones. Finally, they suggested that the density of sorbed CO_2_ is correlated to the mineral matter content of coal seam; in particular, it was observed enhanced CO_2_ density in regions with poor mineral matter. In another study, Mirzaeian and Hall^[162]^ performed *in situ* SANS on a Wyodak coal is a sub‐bituminous coal (Wyodak) by injection of CO_2_ at 25 bar. Their results showed that CO_2_ diffuses into the coal matrix, swells the matrix and possibly creates microporosity by extraction volatile components from coal. Zhang and Liu^[163]^ employed SANS with *contrast matching* to quantify possible gas confinement in the pores of high‐rank anthracite coal. They have also observed highly densified methane molecules in micropores at higher pressures. However, in case of pressurised CO_2_ the results are complex, which could be attributed to many factors such as matrix compression, the local structure of the matrix, swelling induced by adsorption, pore accessibility and the structure of the confined fluid.

Mastalerz et al.^[164]^ have also utilised the SANS/USANS methodology with CO_2_ and CD_4_ complemented by gas (N_2_/CO_2_) adsorption measurements in order to understand differences in the porosity between two Pennsylvanian coal samples and two Upper Devonian‐Mississippian shale samples. Their results showed low accessibility of pores to shale samples. They also demonstrated that accessibility of pores in coal depends on pore size and can differ significantly between coal samples. In addition, the adsorption isotherms on coal samples suggested that higher accessibility corresponds to higher adsorption capacity. They also observed that the increase in depth causes a shift toward smaller pore sizes (within the mesopore and micropore range) in the coal and shale samples. This finding implies that the pore size distribution in both samples may be modified in response to changing depth and/or maturity. Finally, correlations between the total organic matter (OM) content in both coals and shales and their mesopore and micropore volumes (all pores with size <50 nm), as well as their surface area provide an evidence that OM is an important contributor to this pore range. Liu et al.^[165]^ have also carried out *in situ* SANS measurements under incremental pressure (20, 40, 68 bar) to explore the CO_2_ and CD_4_ accessibility to pores for a sub‐bituminous (Hazleton) coal, an anthracite (San Juan) coal and two Marcellus shale samples. The total porosity of all samples is low, varying between 0.25 % and 5.8 %. They found that greater than 75 % of the fraction of accessible porosity for the two shales is accessible to methane, while less than 50 % of the total porosity is accessible to methane for the two coals. This finding indicates that the organic matter pores tend to be disconnected in this nanopore range compared to mineral matter pores. They also observed the variations in the states of CO_2_ and CD_4_ as a function of temperature and/or pressure. Finally they demonstrated that, at a similar pressure, the densification of CD_4_ occurs in larger pores compared to CO_2_.

In another work, Ruppert et al.^[166]^ investigated the pore accessibility to deuterated methane (CD_4_) and heavy water (D_2_O) in two Mississippian Barnett Shale samples by carrying out SANS/USANS measurements. Their results revealed that the total pore size distribution is essentially identical for the two samples at pore sizes larger than 250 nm. However, the smaller pores with a diameter of less than 30 nm appear to be much more accessible to water than to methane. This is evidence that the difference in the connectivity of the same pore to different fluids is controlled by the wettability of the pore surface. Furthermore, this finding indicates that small‐scale heterogeneities in methane accessibility occur in the shale samples, even though the total porosity does not differ. They also proposed that methane condensation in nanopores is not uniform leading to formation of separate nano‐size clusters. Clarkson et al.^[167]^ have also examined the pore accessibility to CD_4_ in a Mississippian Barnett shale sample by *contrast matching* SANS. They found that larger pore sizes (from 600 nm to 4 μm) have lower accessibility to methane compared to smaller pore sizes (from 80 nm to 600 nm). Eberle et al.^[168]^ conducted the first direct measurements of methane density within the inorganic and organic pores in shale by performing *in situ* SANS measurements under vacuum and under pressure with deuterium gas (D_2_), protonated methane (CH_4_) and deuterated methane (CD_4_). D_2_ was utilised to measure experimentally the SLD of the shale matrix because it can be considered as non‐condensed gas. They found that these two pore populations have vastly different methane storage capacities, with the organic pores containing roughly twice the methane density at 21 °C and 338 bar (0.48 g/cm^3^ compared to 0.2343 g/cm^3^, which is the bulk methane gas density). Their results provide an explanation why the sorption capacity of shale scales with total organic content (TOC) and dominates the total storage capacity. Furthermore, classical density functional theory (DFT) calculations confirmed the experimental results and they also showed that this excess density in the organic pores persists to elevated temperatures (100 °C), typical of shale gas reservoir conditions. They concluded that this outcome provides new insight into the hydrocarbon storage mechanisms within these reservoirs. In another study, Neil et al.^[169]^ examined how pressure cycling affects methane behaviour in Marcellus shale nanopores by conducting SANS at room temperature and elevated pressures. In particular, they attempted to link the pressure maxima and the pore size with methane recovery efficiency in order to better understand the mechanism controlling methane transport and recovery. It is well accepted that small nanopores in shales are hosted by relatively soft organic matter (kerogen). Their SANS results showed that methane can penetrate the kerogen at pressures up to 3000 psi (207 bar) and alters its structure causing swelling up to ~2.5 %. When pressure increased up to 6000 psi (414 bar) resulted in a slight decrease in the swelling percentage, despite a continued increase in methane uptake. This behaviour was explained in terms of the mechanical compression, which leads to the deformation of the flexible kerogen matrix, resulting in permanent kerogen matrix deformation by closing pore throats and irreversibly trapping methane clusters in the kerogen pore space. Sun et al.^[170]^ argued that such conditions could cause the density of methane confined to pore sizes of less than 20 nm to increase compared to the density of the bulk gas leading to the formation of methane clusters at the nanoscale. As a result, this mechanism explains that the volume of gas produced in abnormally high‐pressure shale gas reservoirs would be much larger than the pore volume of the shale itself. These findings can have implications for pressure management strategies to maximise hydrocarbon recovery, as well as broad implications for fluid behaviour under confinement.

In another study, Radliński et al.^[114]^ explored the accessibility of pores to methane in overmature middle Devonian Marcellus Shale samples utilising SANS and USANS with *contrast matching*, supplemented by other complementary techniques, such as mercury injection capillary pressure (MICP) and low pressure gas (N_2_ and CO_2_) adsorption. Their observations indicated that the supplied methane at *contrast matching* pressure (500 bar) densifies by a factor from 2.3 to 4.2 when confined in the nanopores, which results in the density of confined CD_4_ varying from 0.78 to 1.43 g/cm^3^, respectively. Even their lower value, 0.78 g/cm^3^, is significantly larger than the value of 0.60 g/cm^3^ estimated by Eberle et al.^[168]^ for micropores (1–2 nm) by loading methane at 345 bar. Moreover, they observed that elevated gas pressure causes formation of high‐density methane nano‐clusters in agreement with Ruppert et al.^[166]^ They demonstrated that these clusters are slightly anisotropic polydisperse discs oriented along bedding plane, about 1–12 nm in diameter and with average thickness of 3.6 nm. Their finding is also in agreement with the study of Neil et al.^[169]^ where, upon methane injection in overmature Marcellus samples, formation of nano‐size clusters was observed as a consequence of the methane trapping in the kerogen structure. Finally, they also addressed the pore anisotropy by utilising samples cut parallel and perpendicular to the bedding. Their results showed anisotropy in the out‐of‐bedding direction suggesting that the degree of anisotropy depends on the pore size. Zhang et al.^[171]^ have also conducted *in situ* SANS measurements with the *contrast matching* method using CD_4_ in order to investigate high‐pressure gas storage mechanisms of three shale samples. The results showed that the quartz‐rich Marcellus shale and Longmaxi shale samples have higher pore accessibility than the Illinois shale sample, which has the highest total organic carbon (TOC). However, the Illinois shale has the highest cumulative porosity and surface area among the measured samples. In addition, the gas storage capacities of the shale samples were estimated and compared with the gas adsorption capacities. It was concluded that TOC has a positive correlation with both the gas adsorption capacity and storage capacity at low pressure. On the other hand, they observed that the Longmaxi shale with the highest pore accessibility and open porosity has the highest gas storage capacity at high pressure. Their results also indicate a lower average adsorbed phase density in open pores than bulk phase density for pressures between 100 bar and the *contrast matching* pressure (∼600–700 bar). In contrast, the average adsorbed methane density in open pores might be higher than the bulk methane at pressures below 100 bar and above the *contrast matching* pressure. They concluded that the final injection pressure, TOC and accessible porosity are essential factors for maximising both methane storage and long‐term CO_2_ sequestration in depleted shale reservoirs.

**Figure 10:**
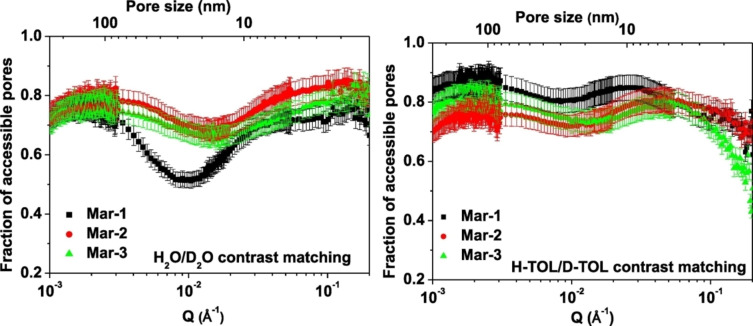
The fraction of accessible pores in three Marcellus Shale samples estimated by: (left) H_2_O/D_2_O *contrast matching* and (right) toluene/d‐toluene (H‐TOL/D‐TOL) *contrast matching*. Reproduced from Ref. [176] with permission from Elsevier.

Apart from the previously discussed neutron study of methane and water in shale by Ruppert et al.,^[166]^ other works have also explored the behaviour of water in shale pores by *contrast matching* SANS and USANS. In a similar work, Gu et al.^[172]^ investigated the water accessibility in a series of Marcellus shale samples. They found that the volume fraction of the pores accessible to water varied between 30 and 52 %. FIB‐SEM observation and quantitative analysis of SANS results showed that clay‐rich shale samples containing low TOC are more accessible to water compared to organic‐rich samples with low clay content; this finding seems to be more apparent in the micropores (<2 nm). They also presented an approach to study the pores in organic matter (OM), which contribute significantly to the total porosity of gas shale and play a major role in defining its storage capacity.^[173]^ Their results indicated that pores in OM larger than 20 nm are accessible to water, despite the fact that OM is generally considered to be hydrophobic. They also concluded that OM pores can account for 24–47 % of the total porosity and occupy approximately 29 % of the OM volume, verifying the significant role of OM pores in controlling shale porosity as well as hydrocarbon storage and transport. Sun et al.^[174]^ further investigated the pore connectivity and water accessibility in clay‐rich Permian Longtan formation shales through *contrast matching* SANS measurements. Their results showed that the majority of pores (from 2 to 200 nm in diameter) are water accessible (87–98 %) in clay‐rich transitional shale. samples. Low accessibility to water was observed at pore sizes of 5–10 nm and 20–30 nm, which may be due to the formation of a water film confined within hydrophobic organic nanopores. Neil et al.^[175]^ utilised H_2_O/D_2_O *contrast matching* SANS at elevated pressures for clay‐ and carbonate‐rich shales from the Permian Basin. Their results indicated that the porosity of the clay‐rich shale was 7.7 %, compared to 0.51 % for the carbonate‐rich shale. However, only 2.6 % of the nanopores in the carbonate‐rich shale were inaccessible to water at 8 kPSI (551 bar) compared to 7.8 % for the clay‐rich shale. The size of the closed pores was estimated to be about 10 nm, probably corresponding to kerogen‐rich pores that store hydrocarbons. Although there were also accessible pores with larger and smaller sizes compared to the closed pore sizes, these pores were closed to water possibly due to the hydrophobic nature of the pore organic matter (OM) host material. Furthermore, they observed that the fraction of closed pores is minimised after 2 kPSI (138 bar) for carbonate‐rich shale and 4 kPSI (276 bar) for clay‐rich shale, respectively. Conclusively, these variations in porosity and pore accessibility could play an important role for the performance of effective hydraulic fracturing operations. Bahadur et al.^[176]^ used *contrast matching* SANS to probe the accessibility of water and toluene in Marcellus shale with various lithofacies. The results suggested that water accessibility depends on type of lithofacy. In particular, the quartz‐rich shale sample exhibits the lowest accessibility compared to the clay‐ and carbonate‐rich samples, respectively. Furthermore, they evaluated the wettability of pores in shale by comparing the pore accessibility to water and toluene. They observed that accessibility to toluene follows that of water; however, pores with size larger than 5 nm are more accessible to toluene in the three samples (Figure 10).

Zhang et al.^[177]^ also utilised *contrast matching* (U)SANS to investigate the multiscale wettability (from μm to nm scale) in four Utica and Bakken shale samples by comparing fluid‐accessible porosity to total porosity for hydrous (water) and organic (n‐decane) wetting fluids. Their results showed that 40–70 % of the pores in the Utica shale samples (mixed carbonate mudstone) were oil accessible, while 34–37 % were water accessible. On the other hand, the Bakken shale samples (mixed siliceous mudstone and carbonate/siliceous mudstone with high TOC content) were less wetted by either oil (less than 23 %) or water (22–36 %). In addition, for both formations, pores less than 3 nm in diameter are not oil accessible (organic matter related) but water accessible (clay tactoids related). In another work, Sun et al.^[178]^ used *contrast matching* SANS to study the pore accessibility to water and toluene in overmature marine shales of China. They demonstrated that the larger pores (pore size >7 nm) of the Wufeng‐Longmaxi shale samples were filled with toluene due to the development of organic pores. On the other hand, more water‐accessible pores were observed for smaller pore sizes (less than 7 nm). This result suggests that the pore volume is perhaps composed of hydrophilic pores related to clay minerals or created by the swelling of clays. Furthermore, when the organic matter (OM) was removed and extracted, toluene could enter the inorganic pores, and water could access the organic pores.

Combination SANS and USANS could be also proved to be an essential tool to examine the oil recovery process from nano‐ to micron‐sized pores in shale. In the following, two (U)SANS experiments have been conducted to investigate *in situ* decane removal from shale by utilising methane as the injectant. In particular, Neil et al.^[179]^ examined the extent of decane saturation and recovery after the end of methane pressurisation in clay‐rich and carbonate‐rich shale samples. The results showed that although clay‐rich shale had a much higher porosity (5.6 %), compared to carbonate‐rich shale (1.2 %), the clay‐rich shale had significantly more decane retention in small nanopores than the carbonate‐rich one. They also found that the majority of these nanopores fall in the range of 3–10 nm in radius, implying that they might hosted by organic shale components, such as kerogen. Another finding was that decane removal could not necessarily be correlated with the degree of decane saturation. This was attributed to the fact that although organic matter‐rich shale had a more significant imbibition of decane and a much higher porosity, only a small percentage of the decane was displaced compared to carbonate‐rich shale. In the other experiment, Neil et al.^[180]^ also utilised decane to saturate two mineralogically distinct shale samples in order to investigate the location of oil in shale nanopores and its retention degree by accomplishing the pressure cycling by CD_4_. They observed significantly more retention of decane in 1.5–10 nm radius pores of both samples, likely indicating that oil is retained within kerogen nanopores. Their findings of this work in conjunction with those of their previous study,^[179]^ suggest that the majority of decane hosted by kerogen nanopores is not recoverable by methane pressure cycling. They concluded that further exploration and improvement of hydrocarbon recovery processes from the kerogen nanoporous matrix is required.

**Figure 11:**
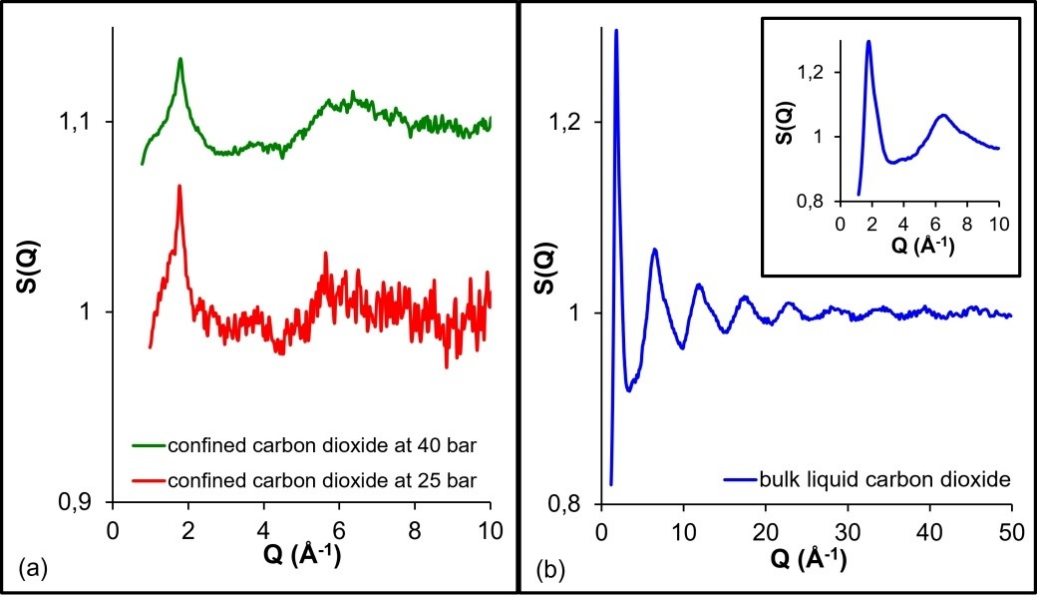
(a) The total scattering structure factors for CO_2_ confined in shale at 25 and 40 bar at 22 °C; the structure factor at 40 bar has been shifted by 0.1 for clarity. (b) The total scattering structure factor for bulk liquid CO_2_ at −43 °C and 12 bar. Inset: Highlight of the low‐*Q* region. Reproduced from Ref. [181], (https://pubs.acs.org/doi/10.1021/acs.est.6b05707), with permission from ACS Publications. Further permissions related to the material excerpted should be directed to the ACS.

In the following studies, combined neutron total scattering and SANS measurements have been carried out on NIMROD instrument (ISIS Neutron and Muon Source). The wide range of length scales in real space (0.1<*d*<310 Å) that can be explored with NIMROD, results in probing simultaneously the structural properties of pore‐confined greenhouse gases as well as their accessibility to micropores (i. e. pores with size less than 2 nm) in shale samples. Stefanopoulos et al.^[181]^ conducted measurements of adsorbed CO_2_ in a Marcellus shale sample (quartz‐rich, from Pennsylvania) along an adsorption isotherm of 22 °C and pressures of 25 and 40 on NIMROD instrument. Additional *in situ* SANS measurements have also been performed at 22 and 60 °C (at the same pressures) on the GP‐SANS instrument at Oak Ridge National Laboratory. The calculated structure factors (from NIMROD experiment) of pore‐confined CO_2_ in shale suggest that at 22 °C the CO_2_ has liquid‐like properties even at pressures as low as 25 bar (Figure 11). The results are in agreement with those of supercritical methane^[168]^ which revealed gas condensation when confined in shale micropores. The results also suggest that by decreasing pore size, pores become increasingly inaccessible to CO_2_; this implies that all pores less than 0.25 nm in radius are inaccessible to CO_2_ (note that the kinetic diameter of CO_2_ is 0.33 nm). Despite the vast numbers of micropores in shale, a majority of micropores are inaccessible to CO_2_, suggesting that this class of pores is unavailable for geologic CO_2_ sequestration. Ruppert et al.^[182]^ further carried out measurements on NIMROD to investigate two Marcellus shale samples (from West Virginia) upon CO_2_ injection at 22 °C and pressures varying between 20 and 50 bar. The results were in excellent agreement with the previous NIMROD experiment,^[181]^ suggesting that during anthropogenic CO_2_ sequestration, the smaller mesopores and all of the micropores in shale will be unavailable sites for storage. Again, the confined CO_2_ had liquid‐like properties at all pressures. The authors also concluded that their study could offer insight into the low recoveries of petroleum during CO_2_‐EOR in many tight sand reservoirs; one reason might be the presence of closed pores (e. g., those that lack pore throats and those that contain condensed CO_2_) that they cannot function as a flow network to disperse CO_2_ through the formation. In another experiment, the same shale samples were pressurised with CD_4_ at 60 °C from 10 up to 65 MPa (the ZAC pressure was 60 MPa or 600 bar).^[183]^ It was found that the quartz‐rich sample adsorbed up to 4 times more CD_4_ compared to the other shale sample, which contains equivalent amount of quartz, carbonate and clay. This indicates that the total available porosity within the quartz‐rich sample was greater, most likely due to increased volume fraction of organic pores. This finding suggests that shale porosity can vary drastically on the meter length scale, at least for formations containing organic matter within the dry gas stage of thermal maturity. The surprising outcome of this study was that the calculated total structure factors of the confined CD_4_ showed little to no evidence for densification of CD_4_ within the sample pores, which is in contrast to previous findings by Eberle et al.^[168]^ and Radliński et al.^[114]^ This was attributed to the contribution of confined CD_4_ from the larger shale meso‐ and macropores, where no densification was expected, obscuring, thus, the signal from CD_4_ confined within smaller micropores.

**Figure 12:**
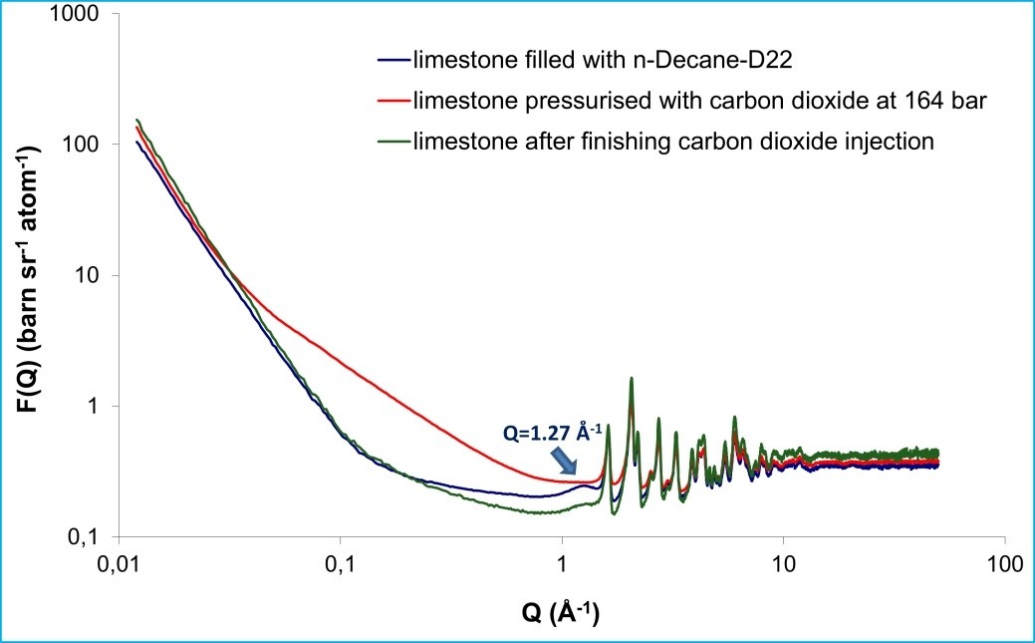
Neutron scattering profiles of limestone sample filled with deuterated n‐decane during (*P*=164 bar) and after finishing the CO_2_‐EOR process at 393 K. Reproduced from Ref. [184] with permission from the Royal Society of Chemistry.

Combination of neutron total scattering and SANS could also provide key insights into CO_2_‐EOR process and CO_2_ sequestration. In a recent study, Stefanopoulos et al.^[184]^ attempted to monitor the CO_2_‐EOR process in real time at the nanoscale by carrying out total neutron scattering (combined with a region of SANS) measurements at NIMROD instrument. This was attained by *in situ* injection of supercritical CO_2_ into a limestone sample loaded with deuterated n‐decane. The results demonstrate directly the decane recovery after the end of the supercritical CO_2_ loading process (Figure 12). Furthermore, it was found that only a small fraction of the smaller mesopores is accessible to CO_2_, suggesting that this class of pores is unlikely sites for geological CO_2_ sequestration. Finally, calculation of the total structure factors elucidated that the confined supercritical CO_2_ is in a densified supercritical state with higher density compared to the bulk phase. Another interesting finding is that that the clustering of supercritical CO_2_ molecules is observed not only at the bulk state but also when confined within the limestone pores.

## 5 Summary and Perspectives

Nowadays, elastic neutron scattering techniques, especially total scattering and (U)SANS (including *contrast matching* SANS) provide powerful research tools to probe both the microstructural features of porous materials (including porosity, pore size and connectivity) and the complex confined fluid behaviour, arising from nanopore confinement effects and fluid interactions with the nanopore walls. The current review presented the basic principles of these techniques with emphasis on fluids confined in porous materials and provided an overview of recent advances in this area, focusing in greenhouse gases confined in geomaterials with applications to CO_2_ sequestration and enhanced oil and gas recovery (EOR, EGR).

In general, thermal neutrons have high penetration ability, high sensitivity to hydrogen (deuterium) and the experiments can be combined with advanced sample environment (high‐pressure sample cells, gas handling equipment, furnaces and cryostats). As a result, the structural properties and the phase behaviour of confined fluids can be revealed under various thermodynamic states by conducting *in situ* neutron total scattering and (U)SANS experiments. In addition, simulation techniques are increasingly being used for the interpretation of small‐angle and total scattering data in disordered and soft matter systems, including confined phases. In this perspective, further neutron scattering experiments need to be carried out using fluids confined in simple and complex porous systems, such as geomaterials and in wide ranges of *P*‐*T* conditions. Ultimately, synergy between experimental results and computer simulations will be very efficient in understanding confinement effects in fluids.

Carbon capture and storage (CCS) could play a key role in combating climate change by preventing CO_2_ from entering the atmosphere. Geological storage involves injecting anthropogenic CO_2_ into rock formations deep underground. In recent years, neutron scattering techniques have become a popular research tool to probe structural properties of geologic materials and confined geo‐fluids. For instance, further understanding of CO_2_ phase behaviour in confined nanopores could provide useful information about the CO_2_ sequestration potential in geologic formations. The ability of neutrons to distinguish open versus closed porosity, via *contrast matching*, is another significant parameter for CO_2_ sequestration. Accessibility of pore space to CO_2_ could also be critical for enhanced oil and gas recovery (EOR, EGR) because a significant portion of hydrocarbons may be stored in closed pores. This means that the efficiency of the recovery potential would also depend on the extent to which the pores can be accessed. Furthermore, *contrast matching* SANS could be further utilised to study the discrepancy in the accessibility of hydrophilic and hydrophobic fluids in pores. The results could quantitatively assess the wettability of rocks and elucidate the correlation between wettability and pore connectivity, which can shed light about both the migration of hydrocarbon fluids and the imbibition of fracturing fluid.

Whilst *in situ* neutron experiments have already been conducted to monitor, in real time, various processes in porous materials, there is still large potential for future developments. As discussed in Sections 2 and 3, when adsorption, neutron total scattering and (U)SANS measurements are combined the structural properties of the confined phase can be revealed during pore filling. Furthermore, neutron total scattering and (U)SANS experiments have been performed to reveal structural transformations of catalytic materials in real time. Section 4 also discussed the first neutron total scattering and (U)SANS experiments to investigate EOR processes in real time using supercritical CO_2_ and CD_4_ as injectants. The experiments provide information about the degree of oil retention, its location in the porous rock matrix, as well as the structure and the accessibility of the injectant. Finally, further *in situ* neutron experiments are required to explore EOR processes in the presence of additives in order to evaluate their performance and provide key insights in the design of optimal CO_2_ sequestration and enhanced gas/oil recovery projects.

## Conflict of Interests

The authors declare no conflict of interests.

6

## Biographical Information

*Dr Konstantinos L. Stefanopoulos holds a PhD from Department of Pure and Applied Physics, University of Salford, UK (1995). He has worked as a research scientist at Berlin Neutron Scattering Center (BENSC), Helmholtz Zentrum Berlin (HZB), Germany. He has also taught at the Hellenic Open University. He is a researcher at National Centre for Scientific Research (NCSR) “Demokritos” (Athens, Greece) since 2006. His research interests are focused on the structural study of nanoporous materials with an emphasis on neutron scattering methods. Furthermore, he utilises in situ neutron scattering techniques for the investigation of structural and thermodynamic properties of fluids confined in nanopores, with applications to carbon dioxide sequestration and enhanced oil recovery. He is collaborating with many International Neutron Scattering Facilities*.

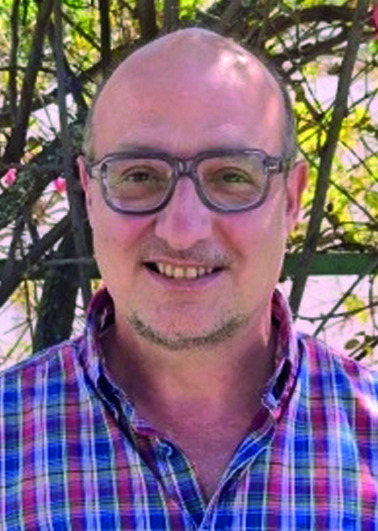

## Data Availability

The data that support the findings of this study are available from the corresponding author upon reasonable request.
